# Novel temporin L antimicrobial peptides: promoting self-assembling by lipidic tags to tackle superbugs

**DOI:** 10.1080/14756366.2020.1819258

**Published:** 2020-09-22

**Authors:** Rosa Bellavita, Annarita Falanga, Elisabetta Buommino, Francesco Merlino, Bruno Casciaro, Floriana Cappiello, Maria Luisa Mangoni, Ettore Novellino, Maria Rosaria Catania, Rossella Paolillo, Paolo Grieco, Stefania Galdiero

**Affiliations:** a Department of Pharmacy, University of Naples “Federico II”, Naples, Italy; b Department of Agricultural Sciences, University of Naples “Federico II”, Portici, Italy; c Center for Life Nano Science@Sapienza, Italian Institute of Technology, Rome, Italy; d Department of Biochemical Sciences, Laboratory affiliated to Pasteur Institute Italia-Fondazione Cenci Bolognetti, Sapienza University of Rome, Rome, Italy; e Department of Molecular Medicine and Medical Biotechnologies, University of Naples “Federico II”, Naples, Italy

**Keywords:** Antimicrobial peptides (AMPs), temporin L analogues, self-assembling, membrane interaction

## Abstract

The rapid development of antimicrobial resistance is pushing the search in the discovering of novel antimicrobial molecules to prevent and treat bacterial infections. Self-assembling antimicrobial peptides, as the lipidated peptides, are a novel and promising class of molecules capable of meeting this need. Based on previous work on Temporin L analogs, several new molecules lipidated at the N- or and the C-terminus were synthesised. Our goal is to improve membrane interactions through finely tuning self-assembly to reduce oligomerisation in aqueous solution and enhance self-assembly in bacterial membranes while reducing toxicity against human cells. The results here reported show that the length of the aliphatic moiety is a key factor to control target cell specificity and the oligomeric state of peptides either in aqueous solution or in a membrane-mimicking environment. The results of this study pave the way for the design of novel molecules with enhanced activities.

## Graphical Abstract

**Figure:**
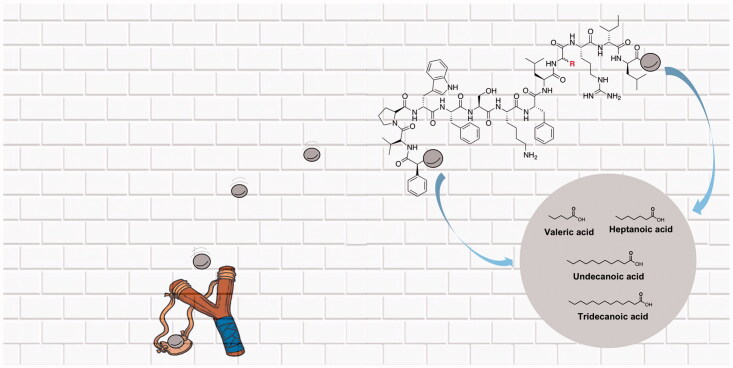

## 1. Introduction

In the last century, antibiotics saved millions of lives and were essential to treat post-operative infections and avoid the progression of infections in immune-compromised patients^1^^,^^2^. However, the growing number of multi-drug resistant bacterial strains, named “superbugs,” has drastically reduced antibiotic efficacy^3^^,^^4^, promoting the rapid development of antimicrobial resistance (AMR), which was recognised in 2015 as one of the biggest global threats to human health by the World Health Organisation (WHO: http://www.who.int)^5^. The nosocomial infections caused by superbugs, including the ESKAPE pathogens (*Enterococcus faecium, Staphylococcus aureus, Klebsiella pneumoniae, Acinetobacter baumannii, Pseudomonas aeruginosa, and Enterobacter* spp.), are the primary cause of human mortality^6^. Among these *K. pneumoniae* has been placed in the list of “priority pathogens,” due to the high diffusion of strains resistant to carbapenems (carbapenemase-producing *K. pneumoniae*), which are considered the last resort for the treatment of infections triggered by resistant Gram-negative bacteria. In this scenario, the discovery of antimicrobial strategies alternative to antibiotics represents a huge challenge essential to overcome and reduce the AMR.

Antimicrobial peptides (AMPs) account for a novel generation of antimicrobial agents^7^^,^^8^. AMPs are produced by most living forms (bacteria, fungi, plants, insects, and vertebrates) and typically consist of variable sequence lengths ranging from 10 to 60 amino acids^9^^,^^10^. Nonetheless, they share common features, i.e. a net positive charge owed to the presence of basic residues such as lysines and arginines, a percentage of hydrophobic residues (about 50%), and an amphipathic nature^11^. Since the primary target of AMPs is the bacterial cytoplasmic membrane, it is troublesome for bacteria to develop a mechanism of resistance making them promising drug candidates. On the other hand, their main drawbacks are cellular toxicity and unfavourable pharmacokinetic properties (protease susceptibility, low *in vivo* half-life, *etc.*)^12^. One of the richest sources of AMPs is represented by the aquatic environment and especially by the amphibian skin of the *Rana* genus^9^. Temporins, isolated from the skin secretions of the European red frog *Rana Temporaria*, is one of the most numerous families in terms of isoforms of AMPs (more than 100)^13^. The isoform L (temporin L, TL), H-Phe-Val-Gln-Trp-Phe-Ser-Lys-Phe-Leu-Gly-Arg-Ile-Leu-NH_2_, is a highly potent AMP with activity against both Gram-positive and Gram-negative bacteria^14^. Recent studies focussed on the mechanism of action of TL showed that hydrophobic residues, in particular the phenylalanine zipper motif, are involved in the initial peptide binding to the Gram-positive cytoplasmic membrane and the stabilisation of peptide aggregates inside the membrane, leading to the formation of large pores and consequential cell death^15^. In the Gram-negative bacterial membranes, the mechanism of permeabilisation appears to be different instead and may not involve membranolytic processes^15^. Unfortunately, TL also kills human erythrocytes at antimicrobial concentration, and earlier structure–activity relationship studies (SAR) showed that this drawback is correlated to its α-helical content^16–19^. In this light, a [Pro^3^,dLeu^9^] analogue of TL, named peptide **1A,** was previously identified, which it was devoid of toxicity to human erythrocytes, but preserved the activity only against the yeast *Candida albicans*^20^^,^^21^. This compound, due to the simultaneous substitution of Gln^3^ and Leu^9^ with proline and d-leucine, respectively, reduced the α-helical content of native TL, and hence the affinity towards eukaryotic membranes. Another lead compound, [Pro^3^,dLeu^9^,dLys^10^]TL (**1B**) derivative, was developed and achieved an enhancement in activity towards Gram-negative bacteria (*P. aeruginosa* ATCC 27853 and *A. baumannii* ATCC 19606)^22^, preserving a modest antimicrobial activity towards Gram-positive bacterial strains. Besides, peptide **1B** did not exhibit significant toxicity against human erythrocytes and keratinocytes at its antimicrobial concentration^23^.

To improve the antimicrobial spectrum activity of peptide **1B** we focussed on the establishment of interactions with the Gram-positive and Gram-negative bacterial membranes to enhance membrane permeabilisation. It is well known that the strength of the interaction and the depth of internalisation into the target membrane are related to the antimicrobial capacity and that a strategy to improve interactions and activities is to increase the hydrophobicity^18^. In this context, a successful approach for increasing the hydrophobicity of charged peptides consists of the addition of saturated fatty acids with different carbon chain length at their N- or C-termini, since long lipid tails facilitate the incorporation into lipid bilayers^24–26^, induce a self-organisation into micelles in the bacterial membrane and improve the membrane permeabilisation^27^^,^^28^. Altogether these effects determine a fluidisation of bilayer and deformation of packing of the phospholipid acyl chain, inducing typically large channel formation and cell death^29^. Moreover, the addition of long hydrophobic lipid tails confers the peptides a self-organisation that makes them also less sensitive to enzymatic degradation and may provide a more effective way to enhance the activity of AMPs, still establishing a promising strategy for the design of antibacterials.

Herein, we reported a library of lipopeptides obtained by the conjugation of saturated fatty acids to both N- and C-terminal regions of **1B** and its parent peptide **1A**, with the aim to reinforce their interaction with the bacterial membrane and to produce a cell specificity, thus to improve the antimicrobial activity towards Gram-positive and Gram-negative bacterial strains, and also to preserve the non-toxicity of peptide **1B** towards eukaryotic cells. The designed compounds were characterised using antimicrobial assays against ATCC reference strains of *S. aureus, K. pneumoniae,* and *P. aeruginosa* and cytotoxicity assay. The mechanism of action of the most promising peptides was studied by fluorescence assays such as Laurdan, Thioflavin T, and leakage assays. Their self-assembling in aqueous solution was calculated by critical aggregation concentration (CAC) and their conformational analysis was performed by CD spectroscopy. Additionally, the most active peptide was preliminarily evaluated for its activity against clinically isolated *S. aureus, K. pneumoniae* and *P. aeruginosa* strains, and finally, its biostability in human serum was assessed. The results are discussed in terms of parameters involved in their potency, specificity and potential to be developed as novel antimicrobials.

## 2. Materials and methods

*N^α^*-Fmoc-protected amino acids used, such as Fmoc-Phe, Fmoc-Val, Fmoc-Pro, Fmoc-Trp(Boc), Fmoc-Ser(*t*Bu), Fmoc-Lys(Boc) and Fmoc-dLys(Boc), Fmoc-Gly, Fmoc-Leu and Fmoc-dLeu, Fmoc-Ile, Fmoc-Arg(Pbf), Fmoc-Phe(4-NO_2_)-OH, all were purchased from GL Biochem Ltd (Shanghai, China). Coupling reagents such as *N,N,N′,N′*-tetramethyl-*O*-(1*H*-benzotriazol-1-yl) uranium hexafluorophosphate (HBTU) and 1-hydroxybenzotriazole (HOBt), as well as the Rink amide resin (0.72 mmol/g of loading substitution), used were commercially obtained by GL Biochem Ltd (Shanghai, China). Fatty acids used, such as tridecanoic, undecanoic, heptanoic, and valeric acids, were purchased by Sigma-Aldrich/Merck. Anhydrous solvents [*N,N*-dimethylformamide (DMF) and dichloromethane (DCM)], 1,3-dimethylbarbituric acid, tetrakis-(triphenylphosphine)palladium(0) [Pd(PPh_3_)_4_], tin(II)chloride [SnCl_2_], 2,2,2-trifluoroethanol (TFE) were purchased from Sigma-Aldrich/Merck. Fmoc-Orn(Alloc)-OH, *N,N*-diisopropylethylamine (DIEA), piperidine, and trifluoroacetic acid (TFA) were purchased from Iris-Biotech GMBH. Moreover, peptide synthesis solvents and reagents, such as *N,N*-dimethylformamide (DMF), dichloromethane (DCM), diethyl ether (Et_2_O), water and acetonitrile (MeCN) for HPLC, were reagent grade acquired from commercial sources (Sigma-Aldrich and VWR) and used without further purification.

Phospholipids: 1,2-dioleoyl-sn-glycero-3-phosphoethanolamine (DOPE), 1,2-dioleoyl-sn glycero-3-phospho-(1′-rac-glycerol) sodium salt (DOPG), and cardiolipin (CL) sodium salt (Heart, Bovine) were purchased from Avanti Polar Lipids (Birmingham, AL, USA), Phosphate-buffered saline (PBS) tablets were bought by Life Technologies Corporation. 8-aminonaphtalene-1,3,6-trisulfonic acid, disodium salt (ANTS) and p-xylene-bis-pyridinium bromide (DPX) were purchased from Molecular Probes. Triton X-100 and the fluorescent probes Nile red, Thioflavin T and 6-dodecanoyl-*N,N*-dimethyl-2-naphthylamine (Laurdan) were purchased by Sigma-Aldrich-Merck. 3–(4,5-dimethylthiazol-2-yl)-2,5-diphenyltetrazolium bromide (MTT) and human serum from human male AB plasma, USA origin, sterile-filtered was obtained by Sigma-Aldrich-Merck.

### 2.1. Peptide synthesis by US-SPPS

#### 2.1.1. Construction of peptide sequences

The synthesis of peptides **1A–5A, 1B–5B, C, D, E** was performed by using the ultrasound-assisted solid-phase peptide synthesis (US-SPPS) integrated with the Fmoc/*t*Bu orthogonal protection strategy^30^. Each peptide was assembled on a Rink amide resin (0.1 mmol from 0.72 mmol/g as loading substitution) as solid support in order to obtain amidated C-termini. In particular, the resin was first placed into a 10 ml plastic syringe tube equipped with Teflon filter, stopper and stopcock, and swollen in DMF on an automated shaker for 30 min at rt. Then, the Fmoc group of the rink amide linker was removed by treatment with 20% piperidine in DMF solution (0.5 + 1 min) by ultrasonic irradiation. The first coupling was carried out by adding a solution of *N^α^*-Fmoc-amino acid, HBTU, HOBt (threefold excess), and DIEA (sixfold excess) in DMF to the resin, thus the resulting suspension was irradiated by ultrasound waves for 5 min. After each coupling and Fmoc-deprotection reaction the resin was washed with DMF (3 × 2 ml) and DCM (3 × 2 ml) and Kaiser or Chloranil tests were employed as colorimetric assays to monitor the progress of the synthesis, used for the detection of solid-phase bound primary and secondary amine, respectively. Subsequent Fmoc-deprotection and coupling steps were performed following the same procedures described above.

#### 2.1.2. Introduction of lipid tags upon peptide sequences 2A–5A, 2B–5B, C, D, E

The introduction of lipid amidated tails for peptides **2A–5A, 2B–5B** was accomplished by reacting the released primary amine in *N*-terminal region with 3 equiv of valeric (**2A** and **2B**), heptanoic (**3A** and **3B**), undecanoic (**4A** and **4B**), tridecanoic (**5A** and **5B**), acids, respectively, HBTU/HOBt (3 equiv) as coupling/additive reagents, in presence of DIEA (6 equiv) in DMF/DCM (1:1 *v/v*), by ultrasonic irradiation for 15 min (Scheme S1, see Supporting Information). Upon filtering and washings (3 × 2 ml of DMF; 3 × 2 ml of DCM) of the resin, the lipidation was ascertained by Kaiser test. Thus, peptides **2A–5A, 2B–5B** were released from the resin and simultaneously cleaved by their protecting groups by using a cocktail of TFA/TIS/H_2_O (95:2.5:2.5 *v/v/v*) at rt for 3 h. Finally, the resins were removed by filtration and crudes were recovered by precipitation with cool anhydrous Et_2_O as amorphous solids. As for the synthesis of peptide **C**, the Fmoc-Phe(4-NO_2_) reagent was used in the replacement of Fmoc-Phe residue in order to give the valeric acid amide in *para* position of the aromatic ring of the Phe^1^ in N-terminal. More specifically, after the elongation of peptide sequence, the resin-bound peptide sequence carrying the Phe(4-NO_2_) was treated with a 1 M solution of SnCl_2_ in DMF and the resulting suspension was gently shaken at rt for 12 h^31^. Such reaction was monitored by LC-MS analysis of the residue obtained from the cleavage of an aliquot of resin [5 mg treated with 1 ml of TFA/TIS/H_2_O (95:2.5:2.5, v/v/v)], and by chloranil test, as colorimetric assay used to reveal resin-bound aromatic primary amines. Once the reduction of the aromatic nitro group was ascertained, the coupling with valeric acid (3 equiv) was performed by adding HBTU (3 equiv), HOBt (3 equiv), and DIEA (6 equiv) to the resin, and the mixture was therefore stirred on automated shaker at rt for 2 h. The LC-MS analysis and chloranil test were repeated to confirm coupling had achieved >80% conversion. Peptides **D** and **E** were otherwise obtained by the introduction of Orn(Alloc) residue at C-terminal. The ornithine allyl protecting group was removed in orthogonal condition with respect to the Fmoc/*t*Bu^32^. Specifically, the resin was treated with a suspension of Pd(PPh_3_)_4_ (0.15 equiv) and 1,3-dimethylbarbituric acid (3 equiv) in DCM/DMF (3:2 *v/v*) and gently shaken for 1 h under argon (Scheme S2, see Supporting Information). The resin was filtered and washed with DMF (3 × 2 ml) and DCM (3 × 2 ml), and the Alloc-deprotection procedure was repeated. After the complete removal was ascertained by Kaiser test and LC-MS, the heptanoic (3 equiv) or valeric (3 equiv) acids were coupled to the δ-amine group of Orn by following the same coupling procedure by ultrasonic irradiation as described above. The resin was filtered and washed with DMF (3 × 2 ml) and DCM (3 × 2 ml) and the lipidation was monitored by Kaiser test.

Finally, the N-terminal Fmoc group was removed and the peptides were treated with a cleavage cocktail, consisting of TFA/TIS/H_2_O (95:2.5:2.5 *v/v/v*), at rt for 3 h, to be released from the resin and cleaved by their protecting groups.

Analytical UHPLC (Shimadzu Nexera Liquid Chromatograph LC-30AD) analyses to assess critical synthetic steps as well as the purity of final compounds **1A–5A**, **1B–5B**, **C**, **D**, **E** were performed on a Phenomenex Kinetex reverse-phase column (C18, 5 μm, 100 Å, 150 × 4.6 mm) with a flow rate of 1 ml/min using a gradient of MeCN (0.1% TFA) in water (0.1% TFA), from 10 to 90% over 20 min, and UV detection at 220 and 254 nm. Purification of peptides **1A–5A**, **1B–5B**, **C**, **D**, **E** was performed by RP-HPLC (Shimadzu Preparative Liquid Chromatography LC-8A) equipped with a preparative column (Phenomenex Kinetex C18 column, 5 µm, 100 Å, 150 × 21.2 mm) using linear gradients of MeCN (0.1% TFA) in water (0.1% TFA), from 10 to 90% over 30 min, with a flow rate of 10 ml/min and UV detection at 220 nm. Final products were obtained by lyophilisation of the appropriate fractions after removal of the MeCN by rotary evaporation. All compounds examined for biological activity were purified to >96%, and the correct molecular ions were confirmed by high-resolution mass spectrometry (HRMS) (Figures S3–S13, Supporting information).

### 2.2. Biology

#### 2.2.1. Bacterial strains and culture

*Staphylococcus aureus* ATCC 25923*, Pseudomonas aeruginosa* ATCC 27853 and *Klebsiella pneumoniae* ATCC BAA-1705 (carbapenemase producer) were obtained from the American Type Culture Collection (Rockville, MD).

Clinical strains of *P. aeruginosa* (*Pa*1, *Pa*2 and *Pa*3), *K. pneumoniae* (*Kp*CR1, *Kp*CR2 and *Kp*CR3), and *S. aureus* (*Sa*1, *Sa*2 and *Sa*3) were isolated from bloodstream or pulmonary infections, and belong to a collection of bacterial strains established between 2010 and 2018 at the Department of Molecular Medicine and Medical Biotechnologies (University Federico II, Naples) for research use. *Pa1, Pa2* and *Pa3* resulted resistant to carbapenems, fluoroquinolones, and gentamicin; *Kp*CR1, *Kp*CR2 and *Kp*CR3 were carbapenem-resistant since KPC carbapenemase producers; *Sa*1, *Sa*2 and *Sa*3 resulted MRSA strains.

Identification was performed by sub-culturing on Tryptic Soy Agar (TSA, Becton Dickinson) and by biochemical characterisation using the Vitek II system (Biomerieux). The antimicrobial susceptibility testing of all isolates was determined using Vitek II system and results were interpreted according to European Committee on Antimicrobial Susceptibility Testing (EUCAST version 9.0, 2019). *P. aeruginosa* was cultured in Brain Heart Infusion broth (BHI, OXOID), *S. aureus* and *K. pneumoniae* were cultured in Tryptic Soya broth (TSB, OXOID), under aerobic conditions at 37 °C, for 24 h on an orbital shaker at 200 rpm.

#### 2.2.2. Mammalian cells

Sheep red blood cells were purchased from OXOID, SR0051D. The human immortalised keratinocytes HaCaT cells (AddexBio, San Diego, CA, USA) were cultured in Dulbecco’s modified Eagle’s medium containing 4 mM glutamine (DMEMg), supplemented with 10% heat-inactivated foetal bovine serum (FBS) and 0.1 mg/ml of penicillin and streptomycin, at 37 °C and 5% CO_2_, in 25 cm^2^ or 75 cm^2^ flasks^33^.

#### 2.2.3. Antimicrobial susceptibility testing

Minimal inhibitory concentrations (MIC) of all the peptides were determined in Mueller–Hinton medium by the broth microdilution assay, following the procedure already described^22^^,^^34^. The compounds were added to bacterial suspension in each well yielding a final cell concentration of 1 × 10^6^ CFU/ml and a final compound concentration ranging from 0.78 to 100 µM. Negative control wells were set to contain bacteria in Mueller–Hinton broth plus the amount of vehicle (ethanol) used to dilute each compound. Positive controls included vancomycin (2 µg/ml), tobramycin (4 µg/ml), gentamycin (4 µg/ml), and imipenem (8 µg/ml). The MIC was defined as the lowest concentration of drug that caused a total inhibition of microbial growth after 16–18 h incubation time at 37 °C.

#### 2.2.4. Cytotoxicity assays

The haemolytic assay was conducted following the procedure described. Aliquots of sheep red blood cells at O.D. of 0.5 (λ = 500 nm) in 0.9% (w/v) NaCl were incubated for 40 min at 37 °C with four different concentrations (ranging from 3.12 to 25 µM) of peptides **1B**, **3B**, **2B** and **C**. Controls were erythrocytes treated with vehicle (water). The samples were then centrifuged for 5 min at 900 × *g*. The amount of haemoglobin released in the supernatant by lysed red blood cells was measured at 415 nm using a microplate reader (Infinite M200; Tecan, Salzburg, Austria) and compared to the complete lysis (100%), obtained by suspending erythrocytes in distilled water^34^^,^^35^. The colorimetric MTT assay was carried out to measure cellular metabolic activity in terms of cell viability^33^^,^^36^. Viable cells are able to reduce, by active mitochondrial dehydrogenases, the yellow salt MTT to purple formazan crystals. Briefly, 4 × 10^4^ HaCaT cells, suspended in DMEMg supplemented with 2% FBS, were plated in each well of a 96-well microtiter plate. After overnight incubation, at 37 °C in a 5% CO_2_ atmosphere, keratinocytes were treated for 24 h with fresh serum-free medium containing the peptides **3B** and **C** at concentrations ranging from 3.12 µM to 25 µM. Afterward, the culture medium was replaced with Hank’s buffer supplemented with 0.5 mg/ml MTT and the plate was incubated at 37 °C and 5% CO_2_ for 4 h. At the end, acidified isopropanol was employed to dissolve the formazan crystals. Cell viability was calculated, with respect to cells not treated with peptide, by absorbance measurements at 570 nm using a microplate reader (Infinite M200; Tecan, Salzburg, Austria)^33^. Colour solution changes are directly related to the number of viable, metabolically active cells.

#### 2.2.5. Plasma stability assay

Human serum from human male AB plasma was acquired by Sigma-Aldrich-Merk. Water and MeCN were obtained from commercial suppliers and used without further purification. Analytical RP-UHPLC was performed on a Shimadzu Nexera equipped with a Phenomenex Kinetex column (C_18_, 4.6 mm x 150 mm, 5 mm) and H_2_O (0.1% TFA) and MeCN (0.1% TFA) as eluents.

Serum stability was evaluated by modification of elsewhere described methods^37^^,^^38^. In particular, the human serum was allocated into a 1.5 ml eppendorf tube and temperature equilibrated at 37 ± 1 °C for 15 min. Peptides **1B**, and **C** were dissolved in sterile water to prepare a stock solution of 2 mM and then mixed with the human serum to make a final concentration of 0.2 mM (90% serum). The resulting reaction solution was incubated at 37 ± 1 °C. Aliquots of the reaction solutions were taken at known time intervals (0, 15, 45, 75, 90, 120 min), subjected to serum proteins precipitation by addition of MeCN (double volume respect to the aliquot). The cloudy reaction sample was cooled (4 °C) for 15 min and then spun at 13000 rpm for 10 min to pellet the precipitated serum proteins. The supernatant was then analysed by RP-UHPLC by using a linear elution gradient from 10 to 90% MeCN (0.1% TFA) in water (0.1% TFA) over 20 min. A flow rate of 1 ml/min was used, absorbance was detected at 220 nm, and the analysis was performed at room temperature.

### 2.3. Critical aggregation concentration (CAC) determination

The solvatochromic fluorescent probe Nile red is widely used to determine the CAC of self-assembling peptides. Nile red shows a blue shift with decreasing solvent polarity. Since Nile red is poorly soluble in water, there is a large preference to partition aggregates, which offer hydrophobic binding sites. Initially, 1 mM methanolic Nile red was prepared. Then, the methanolic Nile red was diluted with water in order to have a final concentration of 500 nM (solution A). The peptides **1B, 2B, 3B, C,** were prepared as follows^39^: peptide stock solutions (0.4 mM) were prepared by dissolving the single peptides in water (**1B, C**) or tetrahydrofuran (**3B**, **2B**) and sonicating for 15 min. Different aliquots were taken to prepare solutions of each peptide at different concentrations (1, 5, 10, 15, 20, 30, 50, 100 and 200 µM). Then, all solutions were diluted with water, sonicated for 15 min and freeze-dried. Finally, the peptide powders were dissolved with the right volume of solution A and allowed to stand in the dark place for 1 h before measurement. Emission spectra for each solution were measured by a Cary Eclipse Varian spectrometer. Spectra were taken between 570 and 700 nm at a slit width of 5 nm, using an excitation wavelength of 550 nm and a 10 nm slit width. The measurements were performed in triplicate. The data were analysed by plotting the maximum emission fluorescence corresponding wavelength (*y*) as a function of peptide concentration (*x*) and fitting with the sigmoidal Boltzmann equation:

$$y=\frac{A1+A2}{1+e^{\left(x-\frac{x0}{\Delta x}\right)}}+A2$$

where the variables *A*_1_ and *A*_2_ correspond to the upper and lower limits of the sigmoid, *x*_0_ is the inflection point of the sigmoid and Δ*x* is the parameter, which characterises the steepness of the function. The sigmoidal plot allows calculating the CAC value at *x*_0_.

### 2.4. Liposome preparation

Large unilamellar vesicles (LUVs) and small unilamellar vesicles (SUVs) consisting of DOPG/CL (58/42 ratio in moles) and DOPG/DOPE/CL (63/23/12 ratio in moles), mimic the Gram-positive membrane and Gram-negative membrane, respectively. LUVs were prepared using the extrusion method as previously described^40^. In particular, first lipid films were prepared by dissolving an appropriate amount of lipids in chloroform and fluorescent probes were added when necessary, then dried under a nitrogen gas stream and freeze-dried overnight. In all experiments, we used a final lipid concentration of 0.1 mM. For fluorescence experiments, buffer was added to dry lipid films and vortexed for 1 h; then the lipid suspension was freeze-thawed 6 times and extruded 10 times through polycarbonate membranes with 0.1 µm diameter pores to obtain LUVs. For circular dichroism measurements, peptide samples in SUVs were prepared as reported in the following protocol^41^. Lipids were dissolved in chloroform and added to an equal volume of peptide solution dissolved in TFE containing appropriate peptide concentration. The samples were vortexed and lyophilised overnight. The dry samples were rehydrated with phosphate buffer 5 mM, pH 7.4 for 1 h and sonicated for 30 min.

### 2.5. ANTS/DPX leakage assay

The ANTS/DPX leakage assay was used to measure the ability of peptide to permeabilise and induce leakage of encapsulated dyes. After lipid films were prepared as previously described, ANTS (12.5 mM) and DPX (45 mM) were dissolved in 2 ml water, were added to lipid films and then lyophilised overnight. The lipid films with encapsulated ANTS and DPX were hydrated with PBS 1X buffer, vortexed for 1 h and then treated to obtain LUVs. The non-encapsulated ANTS and DPX were removed by gel filtration using a Sephadex G-50 column (1.5 cm × 10 cm) at room temperature^42^. To start up the leakage experiment, a 2 mM peptide stock solution was prepared and LUVs were titrated with the peptide. Samples were excited at 385 nm (slit width, 5 nm) and fluorescence emission was recorded at 512 nm (slit width, 5 nm). After the addition of peptide, we evaluated leakage of encapsulated ANTS through a change in fluorescence spectra of ANTS and DPX. In particular, leakage is associated to an increase in ANTS fluorescence at 512 nm. Complete release of ANTS was obtained by using 0.1% Triton X, which caused the total destruction of liposomes. The percentage of leakage was calculated as % leakage =(*F_i_*−*F*_0_)/(*F_t_*−*F*_0_), where *F*_0_ represents the fluorescence of intact LUVs before the addition of peptide, and *F_i_* and *F_t_* denote the intensities of the fluorescence achieved after peptide and Triton-X treatment, respectively.

### 2.6. Membrane fluidity

Membrane fluidity was determined using LUVs containing the fluorescent probe Laurdan^43^. Laurdan was encapsulated into lipid films (0.1 mM) at a concentration of 0.001 mM. After lipid films with Laurdan were lyophilised, hydrated with PBS 1X buffer, pH 7.4, and vortexed for 1 h, they were freeze-thawed 6 times and extruded 10 times through polycarbonate membranes with 0.1 µm diameter pores, obtaining LUVs. The variation of fluidity membrane in presence of peptide was evaluated at 5 and 30 µM, under and above the CAC. The peptide, dissolved in water (2 mM peptide stock solution), was added to LUVs at specific P/L molar ratio and after 10 min, the fluorescence spectra were recorded using a 1 cm path length quartz cell, thermostated at 25 °C. Spectra were corrected for the baseline signal. Laurdan emission spectra were recorded from 400 to 550 nm with λ_ex_ 365 nm in the absence or presence of peptide. Laurdan emission can shift from 440 nm, in the ordered phase, to 490 nm in the disordered phase. The Generalised Polarisation (GP) is a parameter commonly used to quantify the change in the lipid fluidity. It was calculated as GP = (I_440_ − I_490_)/(I_440_ + I_490_), where I_440_ and I_490_ are the fluorescence intensities at the maximum emission wavelength in the ordered (λ_em_ 440 nm) and disordered (λ_em_ 490 nm) phases^43^.

### 2.7. Peptide aggregation

Peptide aggregation in bacterial membrane was assayed using fluorescent probe Thioflavin T (ThT). ThT associates rapidly with aggregated peptides giving rise to a new excitation maximum at 450 nm and an enhanced emission at 482 nm^42^. Lipid films were hydrated with 100 mM NaCl, 10 mM Tris-HCl, 25 µM Tht buffer, pH 7.4 and then treated as described above to obtain LUVs. Each peptide was dissolved in sterile water to prepare a 2 mM peptide stock solution and LUVs were titrated with a peptide concentration of 5, 10, 15, 20, 30, 50 µM. Fluorescence was measured before and after the addition of peptide into the cuvette using a Varian Cary Eclipse fluorescence spectrometer at 25 °C. Samples were excited at 450 nm (slit width, 10 nm) and fluorescence emission was recorded at 482 nm (slit width, 5 nm). Aggregation was quantified according to the equation, %*A*= (*F_f_* − *F*_0_)/(*F*_max_ − *F*_0_)×100, where *F_f_* is the value of fluorescence after peptide addition, *F*_0_ the initial fluorescence in the absence of peptide and *F*_max_ is the fluorescence maximum obtained immediately after peptide addition.

### 2.8. Circular dichroism spectroscopy

CD spectra were recorded at room temperature on a Jasco J-715 spectropolarimeter in a 1 cm quartz cell under a constant flow of nitrogen gas. The spectra are an average of 3 consecutive scans from 260 to 190 nm, recorded with a bandwidth of 3 nm, a time constant of 16 s, and a scan rate of 10 nm/min. Spectra were recorded and corrected for the blank. A solution of 8 µM of the peptide with SUVs consisting of DOPG/CL (58/42 ratio in moles) was prepared, as reported above, and then hydrated with phosphate buffer 5 mM^44^. Instead, a solution of 8 µM of peptide with SUVs consisting of DOPG/DOPE/CL (63/23/12 ratio in moles) was prepared and hydrated with phosphate buffer 5 mM. Each spectrum was converted the signal to mean molar ellipticity.

## 3. Results

### 3.1. Design

To induce self-assembling properties in cationic peptides and influence their interaction with bacterial membranes and consequently their antimicrobial and broad-spectrum activity, we designed a library of lipopeptides by the addition of fatty acids of variable length at the N- and C-termini of peptide **1B** and its parent peptide **1A**. Valeric, heptanoic, undecanoic and tridecanoic acids were linked at the N-terminus of **1A** and **1B** (Table 1), to obtain, respectively, peptides **2A–5A** and **2B–5B**. Moreover, we designed peptide **C**, characterised by an alkyl chain of 5 carbon atom (C-5) in *para* position of the Phe^1^ aromatic ring, but preserving the net positive charge of +4. Besides, we investigated the effects of C-5 and C-7 hydrophobic lipid tails attached to the side chain of the ornithine residue at C-terminus of peptide **1B** achieving peptides **D** and **E**.

**Table 1.** Lipopeptide sequences of [Pro^3^, dLeu^9^, dLys^10^]TL (**1B**) and its reference peptide [Pro^3^,DLeu^9^] (**1A**).

Compound	Sequence
**1A**	H-Phe-Val-Pro-Trp-Phe-Ser-Lys-Phe-dLeu-Gly-Arg-Ile-Leu-NH_2_
**2A**	**CH_3_(CH_2_)_3_CO**-Phe-Val-Pro-Trp-Phe-Ser-Lys-Phe-dLeu-Gly-Arg-Ile-Leu-NH_2_
**3A**	**CH_3_(CH_2_)_5_CO**-Phe-Val-Pro-Trp-Phe-Ser-Lys-Phe-dLeu-Gly-Arg-Ile-Leu-NH_2_
**4A**	**CH_3_(CH_2_)_9_CO**-Phe-Val-Pro-Trp-Phe-Ser-Lys-Phe-dLeu-Gly-Arg-Ile-Leu-NH_2_
**5A**	**CH_3_(CH_2_)_11_CO**-Phe-Val-Pro-Trp-Phe-Ser-Lys-Phe-dLeu-Gly-Arg-Ile-Leu-NH_2_
**1B**	H-Phe-Val-Pro-Trp-Phe-Ser-Lys-Phe-dLeu-dLys-Arg-Ile-Leu-NH_2_
**2B**	**CH_3_(CH_2_)_3_CO**-Phe-Val-Pro-Trp-Phe-Ser-Lys-Phe-dLeu-dLys-Arg-Ile-Leu-NH_2_
**3B**	**CH_3_(CH_2_)_5_CO**-Phe-Val-Pro-Trp-Phe-Ser-Lys-Phe-dLeu-dLys-Arg-Ile-Leu-NH_2_
**4B**	**CH_3_(CH_2_)_9_CO**-Phe-Val-Pro-Trp-Phe-Ser-Lys-Phe-dLeu-dLys-Arg-Ile-Leu-NH_2_
**5B**	**CH_3_(CH_2_)_11_CO**-Phe-Val-Pro-Trp-Phe-Ser-Lys-Phe-dLeu-dLys-Arg-Ile-Leu-NH_2_
**C**	H-Phe(**4-NHCO(CH_2_)_3_CH_3_**)-Val-Pro-Trp-Phe-Ser-Lys-Phe-dLeu-dLys-Arg-Ile- Leu-NH_2_
**D**	H-Phe-Val-Pro-Trp-Phe-Ser-Lys-Phe-dLeu-dLys-Arg-Ile-Leu-Orn(**NHCO(CH_2_)_3_CH_3_**)-NH_2_
**E**	H-Phe-Val-Pro-Trp-Phe-Ser-Lys-Phe-dLeu-dLys-Arg-Ile-Leu-Orn(**NHCO(CH_2_)_5_CH_3_**)-NH_2_

**CH_3_(CH_2_)_3_CO**: valeric acid moiety, **CH_3_(CH_2_)_5_CO**: heptanoic acid moiety, **CH_3_(CH_2_)_9_CO**: undecanoic acid moiety, **CH_3_(CH_2_)_11_CO**: tridecanoic acid moiety.

### 3.2. Antimicrobial activity

The antimicrobial activity of lipopeptides (**1A–5A**, **1B–5B** and **C**, **D**, **E**) was initially assessed against the reference bacterial strains of *P. aeruginosa* ATCC 27853*, K. pneumoniae* ATCC BAA-1705 and *S. aureus* ATCC 25923 by evaluating their minimal growth inhibitory concentration (MIC). The MIC values are shown in Table 2. Peptide **1A** was active against all tested microorganisms with a stronger efficacy towards *S. aureus* (MIC of 6.25 µM), compared to 50 µM for both *P. aeruginosa* and *K. pneumoniae.* Peptide **1B** resulted to be active against all tested microorganisms with a stronger efficacy towards *S. aureus* (MIC of 6.25 µM), compared to 12.5 µM for *P. aeruginosa* and *K. pneumoniae*. Peptides **4A**, **5A**, **4B** and **5B** did not show any antimicrobial effect up to 100 µM, while the best activity was obtained for peptide **3B** on *S. aureus* (MIC of 3.12 µM), and peptide **C** on *K. pneumoniae* and *S. aureus* (MIC of 6.25 µM). In comparison, peptides **2A** and **3A** were inactive against the Gram-negative bacterial strains while a discrete activity was shown against the Gram-positive *S. aureus* (MIC of 12.5 µM).

**Table 2.** *In vitro* antimicrobial activity of compounds **1A–5A**, **1B–5B**, **C**, **D**, **E**.

	Antimicrobial activity (MIC^a^, µM)		
Compound	*P. aeruginosa*	*K. pneumoniae*	*S. aureus*
	ATCC 27853	ATCC BAA-1705	ATCC 25923
**1A**	50	50	6.25
**2A**	>100	>100	12.5
**3A**	>100	>100	12.5
**4A**	>100	>100	>100
**5A**	>100	>100	>100
**1B**	12.5	12.5	6.25
**2B**	50	50	12.5
**3B**	25	50	3.12
**4B**	>100	>100	>100
**5B**	>100	>100	>100
**C**	25	6.25	6.25
**D**	>100	50	25
**E**	>100	50	25
Gentamicin*	4	4	1
Imipenem*	4	>8	0,5
Tobramycin*	4	>4	1
Vancomycin*	NA	NA	2

The minimal inhibitory concentration (MIC) is defined as the concentration of peptide at which 100% inhibition of microbial growth is observed, after 16–18 h at 37 °C. MICs are the average of at least three independent experiments; *For antibiotics, MIC values are expressed as μg/ml. NA: not applicable.

Peptides **D** and **E** were not effective against *P. aeruginosa*, but active against *K. pneumoniae* and *S. aureus* at 50 and 25 µM, respectively; peptide **2B** weakly worked on Gram negative bacteria (50 µM), while a discrete activity was reported on *S. aureus* (12.5 µM). By considering the results here reported, we selected the peptides that showed the best antimicrobial activity both on Gram-positive and Gram-negative bacteria. Thus, peptides **1B** and **C** were further assessed against clinical strains of multidrug-resistant *P. aeruginosa*, carbapenemase producer *K. pneumoniae,* and methicillin resistant *S. aureus* (MRSA). As reported in Table 3 both peptides **1B** and **C** were able to efficiently inhibit the growth of all the tested clinical strains, even though at different MIC values.

**Table 3.** *In vitro* antibacterial activity of peptides **1B** and **C** against multidrug resistant clinical isolates.

	Antimicrobial activity (MIC, µM)								
Peptides	*Pa*1	*Pa*2	*Pa*3	KpCR1	KpCR2	KpCR3	*Sa*1	*Sa*2	*Sa*3
**1B**	12.5	12.5	12.5	12.5	12.5	12.5	12.5	12.5	6.25
**C**	25	25	25	12.5	12.5	12.5	12.5	12.5	12.5
Gentamicin*	>4	>4	>4	1	>8	1	<1	<1	<1
Imipenem*	>4	>4	>4	>8	>8	>8	8	>8	>8
Tobramycin*	4	4	4	2	2	>4	1	<1	1
Vancomycin*	NA	NA	NA	NA	NA	NA	2	1	2

*For the antibiotic, MIC values are expressed as μg/ml; NA: not applicable.

### 3.3. Biocompatibility and selectivity

#### 3.3.1. Cytotoxicity assays

**Figure 1.**
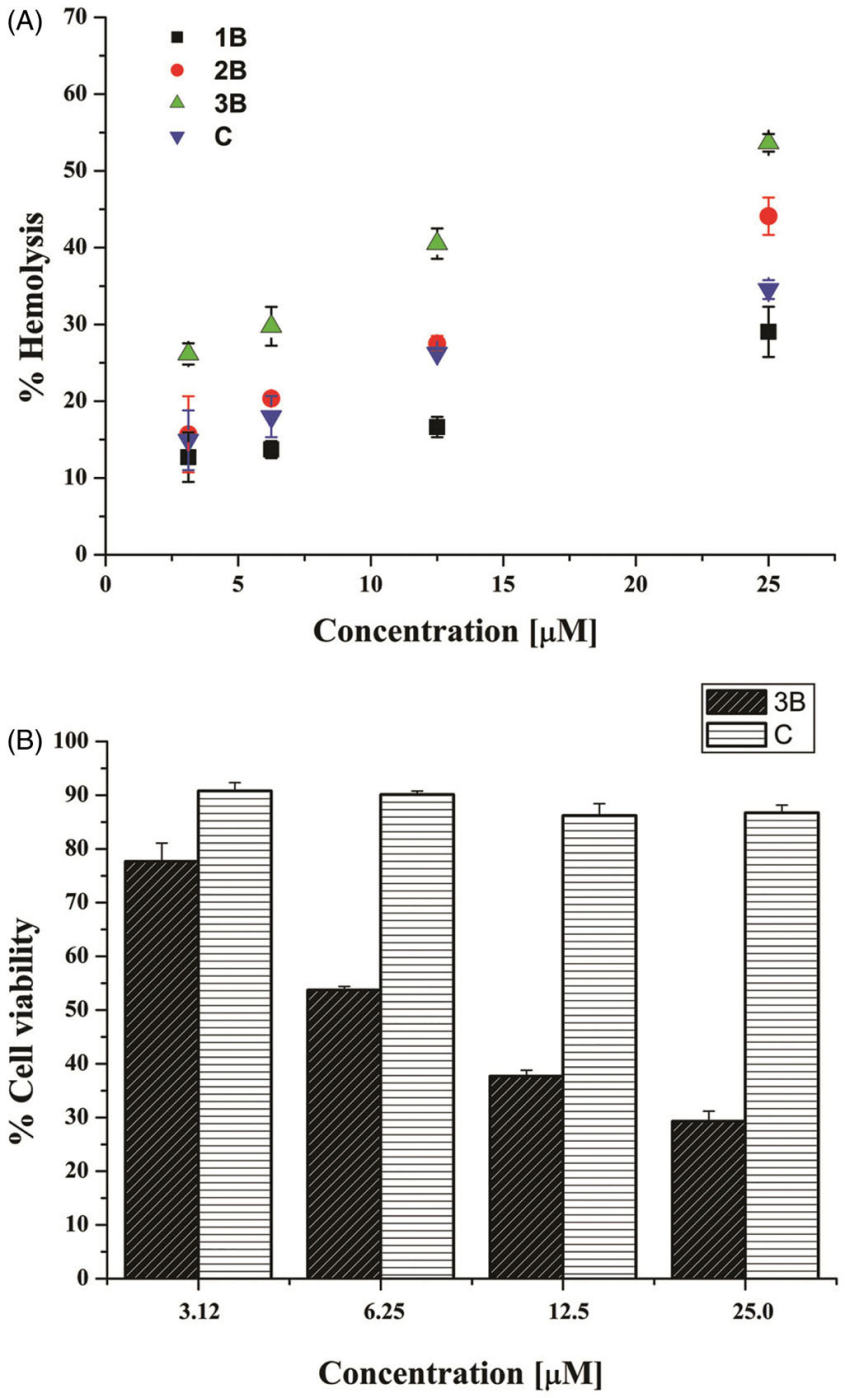
Panel (A) reports the effect of peptides **1B**, **3B**, **2B** and **C** at different concentrations on haemoglobin release from mammalian red blood cells after 40 min of treatment. All data are expressed as a percentage concerning the controls (erythrocytes treated with vehicle) and are the mean of three independent experiments ± standard error of the mean (SEM). Panel (B) reports the viability of peptide-treated HaCaT cells evaluated by MTT assay at 24 h. All data are expressed as a percentage with respect to the untreated control cells and are the mean of three independent experiments ± standard error of the mean (SEM).

The cytotoxic effect of the active compounds, **1B**, **2B**, **3B**, and **C**, was evaluated at short-term against mammalian red blood cells after 40 min treatment at different concentrations. As reported in Figure 1(A), peptides **1B**, **2B** and **C** showed a weak haemolytic activity (lower than 20%) at 3.12 and 6.25 µM. Peptide **3B** showed the highest haemolytic potency (over 50%), especially at the highest concentrations used. Interestingly, compounds **1B** and **C** had a similar haemolytic profile, with a percentage of lysed cells of about 30% at the highest concentrations tested (12.5 and 25 µM). For long-term treatment (24 h), the cytotoxic effect of peptides **3B** and **C** was further investigated by the 3–(4,5-dimethylthiazol-2-yl)-2,5-diphenyltetrazolium bromide (MTT) assay on human keratinocytes (HaCaT cells) (Figure 1(B)). Compound **3B** was confirmed to be harmful causing a strong reduction in cell viability, about 50% already at 6.25 µM. However, note that peptide **C** showed a negligible cytotoxic effect even at the highest tested concentration (25.0 µM), resulting even less toxic than peptide **1B**, already shown in Merlino *et al.* (13.29% cytotoxicity for peptide **C** *vs* 28.22% for peptide **1B**, at 25.0 µM)^22^.

#### 3.3.2. Proteolytic stability

**Figure 2.**
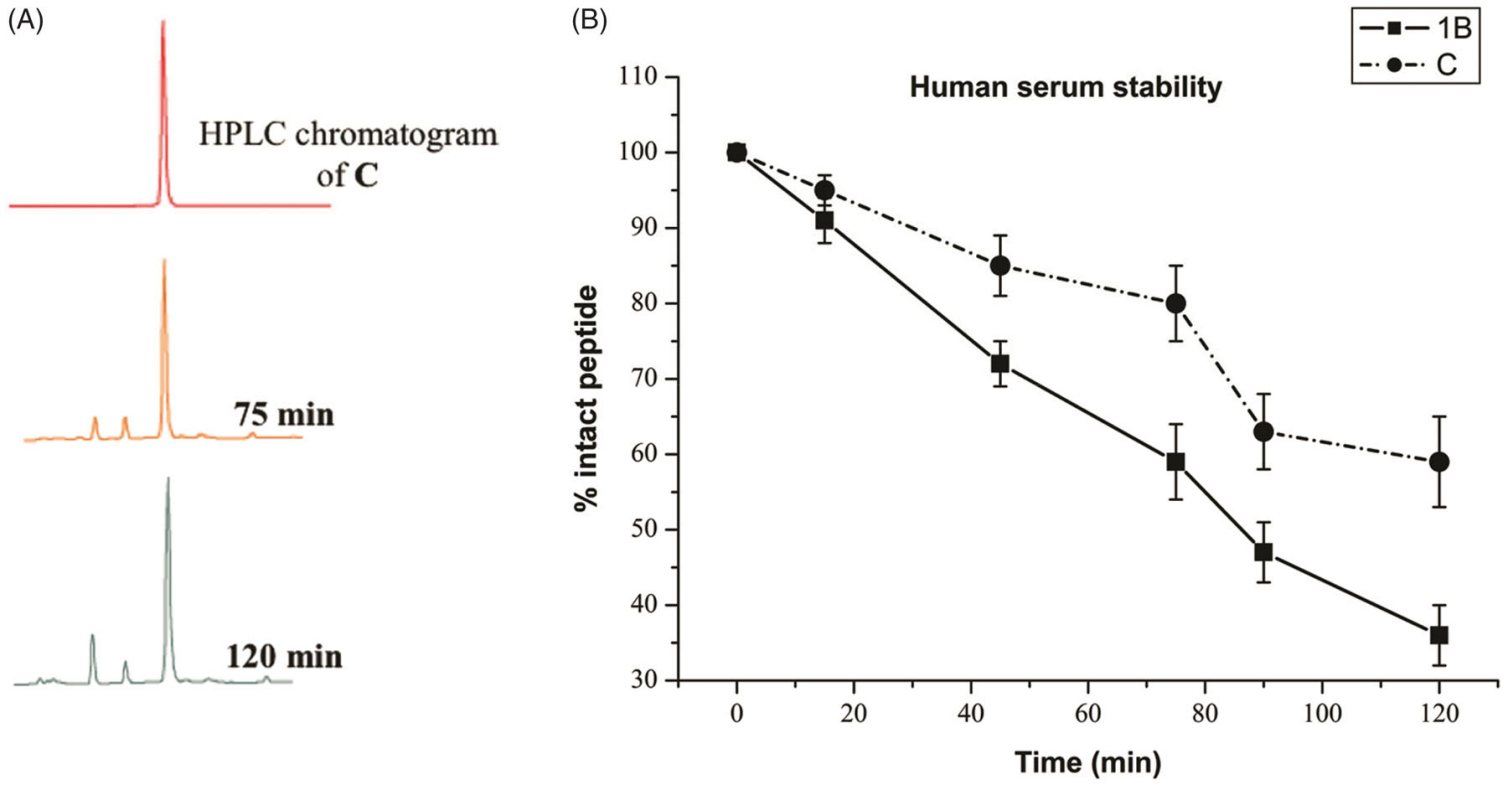
(A) RP-HPLC analysis of peptide **C** in 90% human serum at 37 °C at different times (X, 75, and 120 min). The HPLC profile shows the results of one representative experiment out of at least three independent ones. (B) Percentage of intact peptides **1B** and **C** detected at different time intervals after incubation in 90% fresh human serum (200 µM) at 37 ± 1 °C.

A significant problem of peptides is their protease susceptibility. We probed the proteolytic human serum stability upon peptide incubation with 90% fresh human serum at 37 **±** 1 °C within 120 min and the percentage of intact peptide was calculated by the peak area of the RP-HPLC performed on a Phenomenex Kinetex column (4.6 mm x 150 mm, 5 µm, C18) chromatograms using a linear gradient from 10 to 90% MeCN (0.1% TFA) in water (0.1% TFA) over 20 min (flow rate of 1 ml/min, and absorbance detection at 220 nm). Figure 2 reports the data obtained for peptide **1B** and **C**. As expected, peptide **1B**, our starting sequence is rapidly degraded and cleaved in fragments, notwithstanding the presence of D amino acids. Chemical modifications in the sequence and formation of the self-assembled structures can significantly modify enzymatic degradation rates. As a matter of fact, we observed that peptide **C** is stable up to 1 h (Figure 2); while we observed partial degradation starting from 80 min even though a significant part of the active peptide is present. Clearly, the further addition of the hydrophobic tail in **C** and the formation of supramolecular assemblies can be used as a viable strategy to reduce protease susceptibilities of AMPs.

### 3.4. Peptide assembly

#### 3.4.1. Critical aggregation concentration (CAC)

Nile red was used in a fluorescence assay to determine the ability of the designed peptides to self-assemble. The poor solubility of Nile red is responsible of its preference for hydrophobic binding sites and thus of a blue shift and hyperchromic effect. The change in the emission signal allows to ascertain the formation of the aggregates as well as to determine the concentration at which the aggregate forms (CAC).

**Figure 3.**
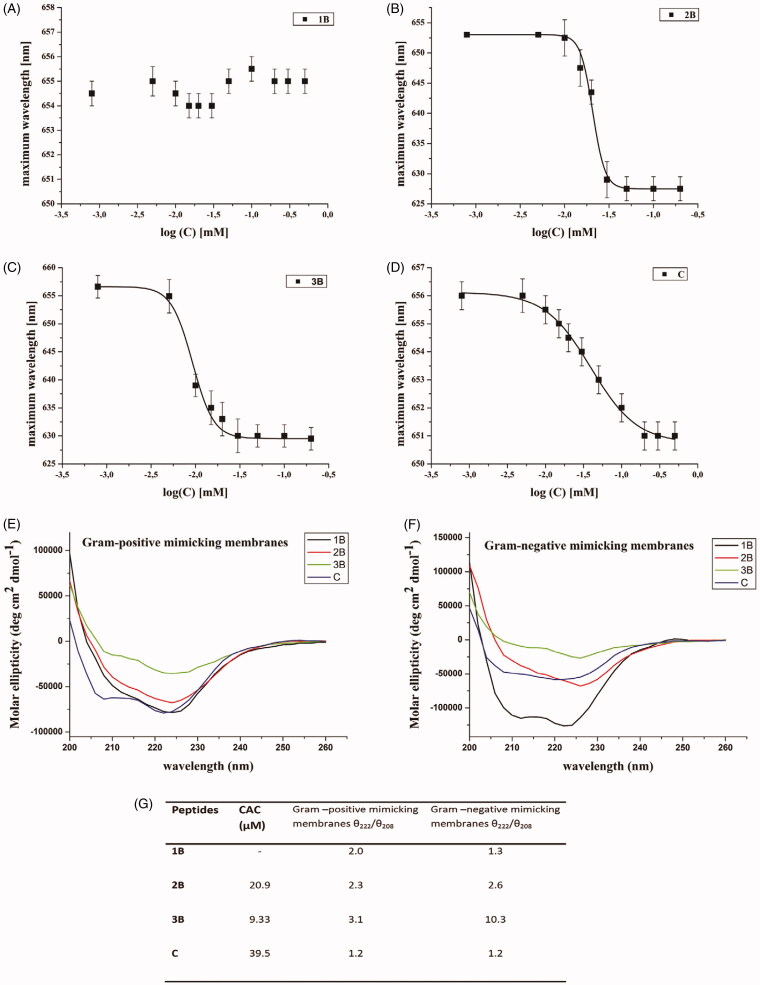
Wavelength corresponding to the maximum fluorescence emission of Nile red was plotted as a function of the concentration of: **1B** (A), **3B** (C), **2B** (B), **C** (D) to determine their CAC. The measurements were repeated three times. Panels (E, F) report the conformational characterisation of the four peptides by CD spectroscopy in membranes mimicking Gram-positive (E) and Gram-negative (F) membranes. Panel (G) reports the critical aggregation concentration (CAC) calculated by a fluorescence assay with the fluorophore Nile red and the ratio of the ellipticities at 222 and 208 nm, which discriminates between monomeric and oligomeric states of helices for peptides **1B**, **2B**, **3B** and **C**.

In Figure 3(A–D), the experiments performed at pH 7 (physiological condition) for all peptides are reported. In particular, the wavelength of Nile red maximum fluorescence emission is reported as a function of the concentration of single peptides. Figure 3(G) summarises the CAC obtained for each peptide.

For peptide **1B** (Figure 3(A)), we were unable to observe any blue shift, indicating that this peptide is unable to aggregate under the range of concentrations investigated. On the contrary, the presence of lipids was sufficient to confer the hydrophobic driving force to promote peptide aggregation.

The modified sequences containing the valeric (**2B**) and heptanoic (**3B**) acids at the N-terminus showed a significant ability to aggregate with a CAC, respectively, of 20.9 and 9.33 µM (Figure 3(G)). The CAC of compound **C** is higher (39.5 µM) indicating that the peptide is still able to aggregate but the presence of the positive charge on the N-terminal amino group reduced its aggregating ability (Figure 3(G)).

### 3.5. Secondary structure

The molecular conformation of the peptides was investigated by far-UV CD spectroscopy, which is widely exploited for the determination of the secondary structure and for studying the formation of peptide assemblies in solution (Figure 3(E,F)). In fact, changes in secondary structures compared to monomers often characterise self-assembly processes. We here determined the secondary structure in liposomes mimicking the Gram-positive and Gram-negative membranes at a concentration below the CAC obtained in aqueous solution in order to better understand if peptides that were not aggregated in water solution could self-aggregate when in the membrane environment. The data obtained clearly demonstrate that the peptides were monomeric with a random coil conformation in aqueous solution (data not shown). Moreover, they showed a significant ability to aggregate in membrane mimetic environments with a high tendency to give helical aggregates in both Gram-positive and Gram-negative membranes. In fact, we observe a helical conformation in Gram-positive membranes with two negative bands at about 208 and 222 nm. The visual analysis of the spectra clearly indicates the presence of aggregates. To establish whether we were observing oligomerisation processes that likely occur through self-assembly in the experimental condition used for our assays we determined the ratio of the ellipticities at 222 and 208 nm (Figure 3(G)), which helps in discriminating between monomeric and oligomeric states of helices^43^. In our spectra, the ratio θ_222_/θ_208_ is always greater than 1.0, indicating an α-helical conformation in its oligomeric state; for a monomeric state, the ratio θ_222_/θ_208_ would have been lower than 0.8^44^.

Moreover, we performed experiments after centrifugation of the samples to get rid from the solution of eventually precipitated peptides and as expected we did not observe any effect on the spectra. One of the main features of peptides able to enter the bilayer is the change of conformation from random to helical^4^.

### 3.6. Membrane interactions

Liposomes containing DOPG/CL (58/42) and DOPG/DOPE/CL (63/23/12) mimicking Gram-positive and Gram-negative membranes were prepared and used for membrane interaction studies.

**Figure 4.**
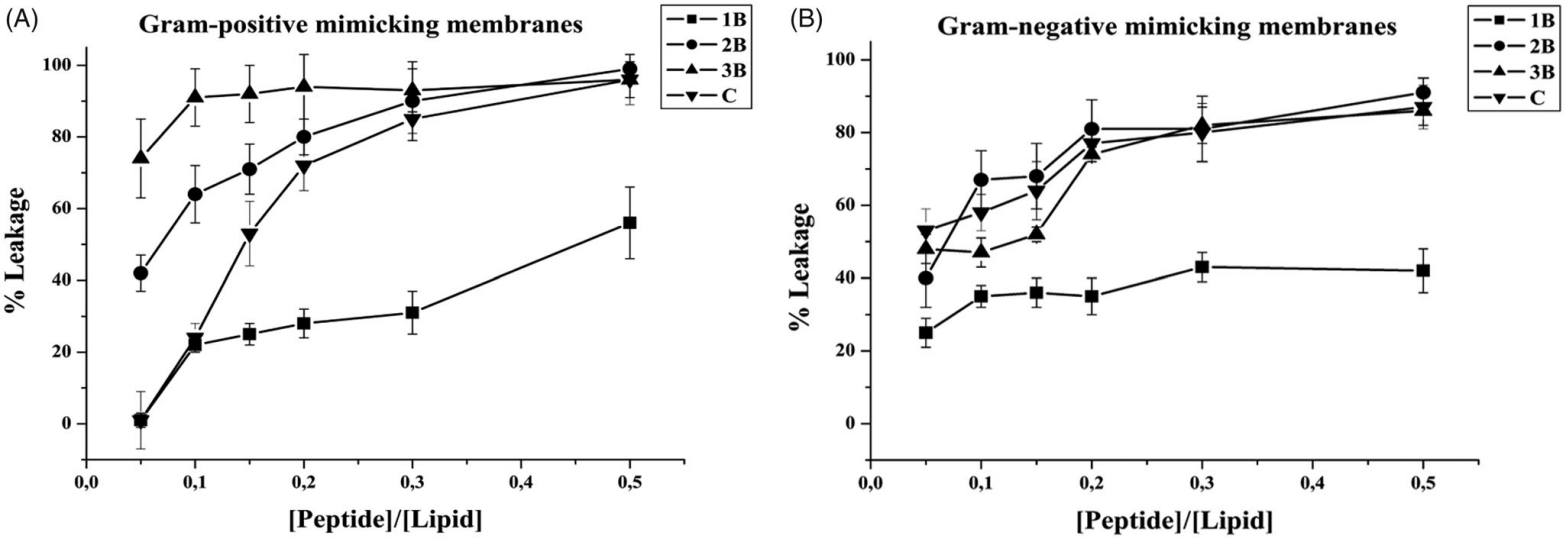
Peptide-promoted membrane leakage of compounds **1B**, **3B**, **2B**, **C** in LUVs mimicking Gram-positive (A) and Gram-negative (B) membranes. The percentage of leakage is reported as a function of the peptide/lipid ratio and each trace represents an average of three independent experiments.

The ANTS/DPX assay was exploited to determine eventual leakage of liposomes mimicking Gram-positive and Gram-negative membranes; indeed, in both tested conditions, we observed a significant leakage with all peptides, supporting pore formation as the primary mechanism of toxicity against bacteria. In particular, we observe higher leakage in presence of Gram-positive membranes; thus, membrane leakage resulted to be strongly dependent on the content of negatively charged lipids. This is in line with the biological data showing a greater activity against *S. aureus* (Figure 4). These data indicate that leakage is involved in the antimicrobial mechanism of these molecules.

Leakage events are typical of AMPs; nonetheless, in order to have promising molecules to be developed for further applications it is important to have membrane perturbation not accompanied by haemolysis. The peptides **2B** and **3B** showed the highest leakage ability but also the highest haemolysis; on the contrary, **1B** presents the lowest leakage ability and its percentage of haemolysis is similar to **C**. The peptide **C** presents an intermediate ability to produce leakage of both Gram-positive and Gram-negative membranes and low haemolysis up to 25 µM. At this concentration, it is also below the CAC, indicating that it is not already aggregated when in contact with the membranes. **2B** and **3B** having lower CACs are likely already aggregated when they induce the leakage of the membrane thus being also haemolytic.

### 3.7. Membrane fluidity

LUVs fluidity before and after the addition of **1B**, **2B**, **3B**, **C** peptides was obtained using the fluorescent probe Laurdan^43^^,^^45^. Laurdan changes its emission when inserted in the gel phase membranes (440 nm) or in the liquid phase membranes (490 nm). The Generalised Polarisation (GP) parameter, which is commonly used to quantify the change in the lipid fluidity, can be calculated from the emissions at 440 and 490 nm. LUVs mimicking Gram positive and Gram negative bacterial membranes were used. The emission spectra clearly indicate the presence of more fluid membranes at 25 °C; the reproducibility of the spectra after 24 h (data not shown) further supported that LUVs were stable and not leaky in the condition used for the experiments. The GP parameter allowed to quantify the effect of the peptide (Table 4). The GP parameter of the membranes mimicking Gram positive bacteria at 25 °C increased significantly for all tested peptides at 30 µM, indicating a shift towards more ordered membranes. Interestingly, **1B** and **C** provided the highest increase. The fluidity of the membranes in the presence of peptides at 5 µM was not modified significantly, except for peptide **C**.

**Table 4.** Membrane fluidity evaluation using the generalised polarisation (GP) value calculate as GP =(I_440_−I_490_)/(I_440_+I_490_).

Compound	Unloaded LUVs	LUVs + 5 µM Compound	LUVs + 30 µM Compound
Gram negative mimicking membranes			
** 1B**	−0.07	0.09	0.11
** 2B**	−0.07	−0.05	0.03
** 3B**	−0.07	−0.07	0.07
** C**	−0.03	0.07	0.15
Gram positive mimicking membranes			
** 1B**	−0.18	−0.06	0.26
** 2B**	−0.18	−0.14	−0.02
** 3B**	−0.18	−0.18	−0.11
** C**	−0.23	−0.14	0.18

For the Gram negative mimicking membranes, we observe the same trend although we obtain fewer modifications.

### 3.8. Peptide aggregation

**Figure 5.**
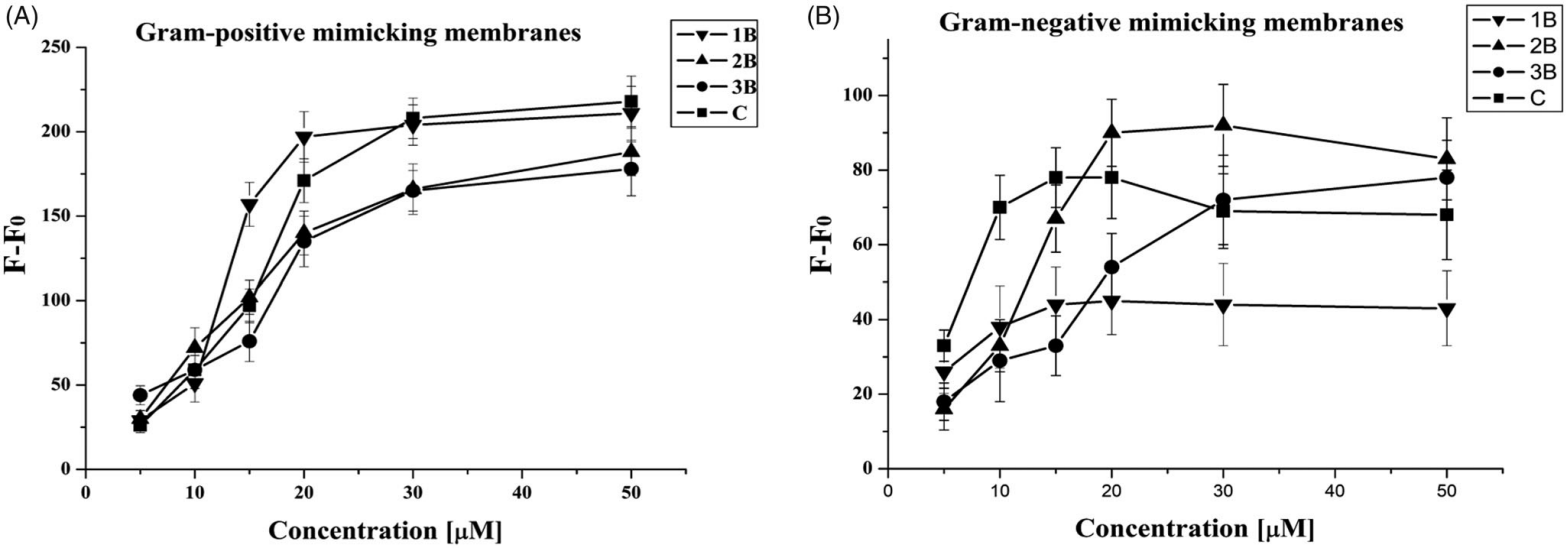
ThT aggregation as a function of the peptide/lipid ratio of **1B**, **3B**, **2B**, **C** in liposomes mimicking Gram-positive (A) and Gram-negative (B) membranes.

The Thioflavin T (ThT) experiment was used to determine the peptide aggregation state in membrane^41^; the results obtained are reported in Figure 5. We observe a dramatic increase of fluorescence as a function of concentration for all peptides, indicating a progressive phenomenon of aggregation in LUVs. In particular, at low peptide/lipid ratios, peptides **1B** and **C** produced a drastic increase of fluorescence, indicating that both peptides oligomerise significantly in liposomes. Peptides **3B** and **2B** showed a lower aggregation at low peptide/lipid ratio while enhanced aggregation was observed at higher ratios as revealed by an increased fluorescence. These results are in agreement with those obtained in the other experiments; in fact, **2B** and **3B** are already aggregated in aqueous solution and thus they aggregate in membranes at a higher concentration as it is likely that they need first to disaggregate in the move between the two environments.

## 4. Discussion

The activity of AMPs in terms of their capacity to permeabilise the membrane of Gram positive and Gram negative bacteria is dependent on different equilibria which take place both in the aqueous and in the lipid milieu. The initial electrostatic interactions between AMPs and negatively charged membranes of bacteria leads to an increased peptide concentration on the membrane surface that favours the membrane penetration through several possible mechanisms such as the carpet, barrel-stave and toroidal pore mechanisms^8^. After the initial electrostatic interaction, the membrane association is driven by the hydrophobic effect; thus, when designing novel sequences with antibacterial activity, hydrophobicity must be finely tuned to optimise activity and selectivity^18^^,^^45^. Molecules with low hydrophobicity are often non-membrane active because their affinity for membranes might be insufficient and/or they may locate too superficially in the bilayer to perturb the surface tension and lead to the formation of pores; however, molecules with a too high hydrophobicity may cause toxicity, because binding to eukaryotic cell membranes becomes significant and haemolysis is observed together or more than antibacterial activity^46^. The increased hydrophobicity may determine a deeper embedding of the peptide inside the lipid bilayer with a reduction in the electrostatic interactions with negatively charged head-groups^47^. Overall, selectivity and specificity can be finely adjusted modifying the ratio between hydrophobicity and hydrophilicity. Furthermore, conformational equilibria may also affect activity modulating the effective hydrophobicity, because they influence the degree to which the hydrophobic side chains are exposed to the water milieu^47^. For instance, disrupting a perfectly amphipathic α-helix through the insertion of helix-breaking residues or polar residues increases peptide selectivity, by reducing the hydrophobic driving force for membrane binding. This was observed for several TL analogues which showed greater activity when the helical propensity was affected^18^. Another key point is represented by the ability of most AMPs to self-assemble into fibrillar amyloid-like nanostructures when located inside the bacterial membrane where they should exert the membrane lytic activity.

Much research is focussed on membrane interactions of monomeric AMPs and how to control lipid affinity to obtain selective destruction of bacterial membranes^48^^,^^49^. However, growing shreds of evidence have shown that AMPs self-assembly offers great opportunities to modify the ratio between hydrophobicity and hydrophilicity and thus antimicrobial activity and selectivity^50^. Nevertheless, aggregation of amphiphilic peptides decreases their effective hydrophobicity masking the apolar moieties from the aqueous phase and membrane binding is reduced. Moreover, in model membranes mechanisms that are present in cellular studies and that could affect selectivity through aggregation, are likely absent; self-assembled AMPs might be ineffective in crossing the LPS layer or the cell wall, and thus in reaching the plasma membrane of bacteria while being still able to interact with the unprotected membrane of host cells. These drawbacks are responsible for the fact that the development of self-assembling peptides with antibacterial activity has not been widely exploited.

Recently, several studies have been carried out on self-assembling materials such as diphenylalanine to provide model systems for the development of antibacterial agents; these systems although efficient in fighting the infection are difficult to develop further because of toxicity issues. A challenging alternative is to engineer AMPs and tune their self-assembling ability favouring aggregation in membrane environments but not in aqueous media to enhance their activity while reducing their toxicity, establishing an innovative design principle for the development of antibacterial materials. Modifications of native sequences may also aid in increasing the stability and half-life of peptides while augmenting and controlling their local concentration.

The spontaneous organisation of molecules into ordered aggregates through supramolecular interactions, known as self-assembly, can be achieved by the rational design. In this context, lipopeptides represent an interesting approach and can be exploited to increase hydrophobicity, favour self-assembly, and boost membrane binding to strongly enhance antimicrobial activity and reduce toxicity against human cells. Residues containing long acyl chains can insert into lipid membranes despite the composition of the latter and induce non-selective, membrane destabilisation. It was also previously shown that single lysine residue attached to palmitic acids are not active, which further supports the view that activity is not only dictated by hydrophobicity but also requires a specific peptide sequence^51^; moreover, it is clear that also the organisation of the lipopeptides in solution and when bound to specific membranes is crucial for activity^51^. The lack of selectivity makes many lipopeptides toxic and consequently restricts their use to critical conditions for which other antibiotics are ineffective rendering important to finely tune also the positive charges of the designed compound.

In this study, we have investigated different aspects related to the antibacterial activity of TL derivatives both in water solution and in membrane mimetic environment.

In an attempt to augment the activity against bacteria and to decrease cytotoxicity, lipidated molecules were produced. Valeric, heptanoic, undecanoic and tridecanoic acids were linked at the N-terminus of **1A** and **1B**, obtaining peptides **2A–5A** and **2B–5B** (Table 1). Lipidated analogues of parent peptide **1A** (**2A–5A**) resulted in less active than those obtained from peptide **1B (2B–5B** and **C**) against both Gram-positive and Gram-negative bacteria (Table 2). Thus, we compared compounds obtained from peptide **1B** and lipidated at the N- or at the C- terminus; we investigated the effects of C5 and C7 hydrophobic lipid tails attached on the side chain of an ornithine residue at C-terminus of peptide **1B**, achieving peptides **D** and **E** (Table 1). C-terminally modified peptides clearly showed reduced activity.

Peptide **3B**, an analogue of peptide **1B** with the C7 hydrophobic lipid tail at the N-terminus showed improved activity against *S. aureus*, but had strong haemolytic activity at its antimicrobial concentration as shown in Figure 1. Based on biological results, we clearly evidenced that long hydrophobic tails caused the non-selective binding to the cell membranes and consequently stronger haemolytic activity. Our results are in agreement with those obtained by other groups and showing that the length of the aliphatic chain is key for peptide activity and organisation^52^.

We also probed the effect of the positive charge on activity and found that a reduction of net positive charge from +4 to +3 compared to peptide **1B** resulted in a loss of activity on Gram-negative strains. Peptide **C,** characterised by an alkyl chain of 5 carbon atoms (C5) in *para* position of the Phe^1^ aromatic ring, was designed to simultaneously preserve both the net positive charge of +4 while adding the alkyl chain and the low haemolytic activity like peptide **1B** (Figure 1). Peptide **C** was able to preserve the same activity of **1B** on *S. aureus*. A comparison among peptides **1B**, **2B**, **3B,** and **C**, clearly showed that peptides **2B** and **3B** display antibacterial activity at higher concentrations compared to **1B** and **C**. The comparison of the biological data obtained for peptides **2B** and **C** clearly show that with a C5 alkyl chain the compound **C** that preserves the net positive charge of +4 is more active against Gram-negative bacteria (Table 1).

Moreover, *P. aeruginosa* and *K. pneumoniae* are among the main pathogens causing hospital-acquired infections and frequently show resistance profiles which reduce greatly the therapeutic options. MRSA infections are also increased in community and hospital settings in the last two decades. Drugs used against MRSA, such as glycopeptides, daptomycin, linezolid, as well as fifth-generation cephalosporins, are not effective against Gram-negative bacteria, so the combination of multiple drugs is needed for empirical infection treatments. Thus, peptides **1B** and **C** were also tested against carbapenem-, fluoroquinolones-, and gentamicin-resistant *P. aeruginosa*, carbapenem-resistant *K. pneumoniae*, and MRSA clinical strains. The two peptides showed good activity against the clinical strains tested, with MIC values very close to those reported for reference strains (Table 3).

The tendency to aggregate in water usually determines a significant reduction in antibacterial activity, which may be attributed to a reduced diffusion of the aggregates through the cell wall, due to their large size in comparison to the corresponding monomers as was previously demonstrated for other peptide sequences^44^^,^^53^. In particular, self-association can affect the water-membrane partition equilibria by shielding the hydrophobic residues from the aqueous phase, thus reducing the hydrophobic driving force for the association to membranes.

We thus decided to probe the ability of these four peptides to self-assemble in aqueous solution and their mechanism of interaction with model membranes mimicking Gram-positive and Gram-negative bacterial membranes in order to clarify from a biophysical point of view if self-assembling could have an impact on activity and could be exploited to tune their efficacy.

Peptides **2B** and **3B** aggregate at low concentrations in aqueous solutions (CAC 20.9 µM and 9.33 µM, respectively) compared to **C** (CAC 39.5 µM) and **1B**, which is not aggregated at the concentration tested (Figure 3(A–D)). The presence of aggregates already in aqueous solutions reduces the ability to interact with the membrane bilayer and thus the antibacterial activity. In fact, all peptides are active at a concentration below their CAC and thus are not aggregated in aqueous solution at the tested concentrations. This means that antimicrobial activity is lost when above the CAC, indicating that solution self-assembly inhibits peptide membrane binding and antimicrobial effect.

At this point, we wanted to prove their ability to oligomerise inside the membrane, which is another key parameter of the interaction of AMPs acting on the membranes of bacteria. We clearly observe that all peptides can oligomerise in the membrane (Figure 3(G)) with the process taking place at lower peptide/lipid ratios for peptides that are monomeric in aqueous solutions (**1B** and **C**). We thus hypothesised that oligomerisation is a key parameter to aid the interactions with the membrane for AMPs but this process has to take place inside the membrane and any oligomer already present in the aqueous solution may prevent the interaction. The ThT assay allowed us to understand that our compound presents a greater interaction with Gram-positive membranes (see *F*−*F*_0_ values that are higher for Gram-positive mimicking membranes) and in particular the greatest aggregation in the membrane is observed for compound **C** (Figure 5).

We thus analysed the effect of the peptides on the stability of the bilayer. The fluidity of the bilayer was analysed below and above the CAC and we found that in membranes mimicking Gram-positive bacterial membrane at 25 °C, the parameter GP, increased significantly for all tested peptides at 30 µM, indicating a shift towards more ordered membranes (Table 4). Interestingly, **1B** and **C** provided the highest increase. Only peptide **C** was able to modify significantly the membrane at 5 µM. For the Gram-negative mimicking bacterial membranes, we observe the same trend. It is interesting to note that eukaryotic membranes are more ordered than bacterial membranes, which lack cholesterol; the most active peptides are those which cause a shift towards more ordered membranes. Likely, the membrane composition of the bacterial membrane which is characterised by the presence of lipids with head groups smaller than the tails favours a concave more disordered shape; the presence of a threshold concentration of the peptides will cause a release of the curvature through membrane defects or pores.

Usually, AMPs assume different conformations and aggregation states in aqueous solution and when bound to membranes. Thus, conformational equilibria play a key role in the activity, modifying the degree to which the hydrophobic side chains are exposed to the water phase; therefore, we probed the secondary structure of peptides **1B**, **2B**, **3B**, **C** in aqueous solution and in liposomes mimicking the membranes of Gram-positive and Gram-negative bacteria. Previous studies in membrane mimetic environments such as micelles and SDS showed that native TL tends to assume helical structures and modifications aiming at reducing the helical content were responsible for lower haemolysis. Here, we observe a clear tendency to oligomerise for all peptides in liposomes mimicking both environments. It is interesting to note that the secondary structure of the oligomerised compounds is different; while we detect a clear molecular helical aggregate for Gram positive mimicking liposomes, we observe β-aggregates in Gram-negative environments (Figure 3(E–G)).

Aggregation may reduce effective hydrophobicity reducing also the affinity for eukaryotic cells and thus toxicity; likely, the local release of a high peptide concentration at a single site in the bacterial membrane will enhance activity. Being the bacterial membranes lacking the cholesterol are more fluid and this feature may determine the higher efficiency of peptides in binding to bacterial membranes.

Therefore, peptide aggregation or ordered self-assembly might positively effect both pharmacokinetic and pharmacodynamic proprieties of AMPs; for example, it might reduce effectively their susceptibility to proteolytic degradation, increasing their half-life.

## 5. Conclusion

**Figure 6.**
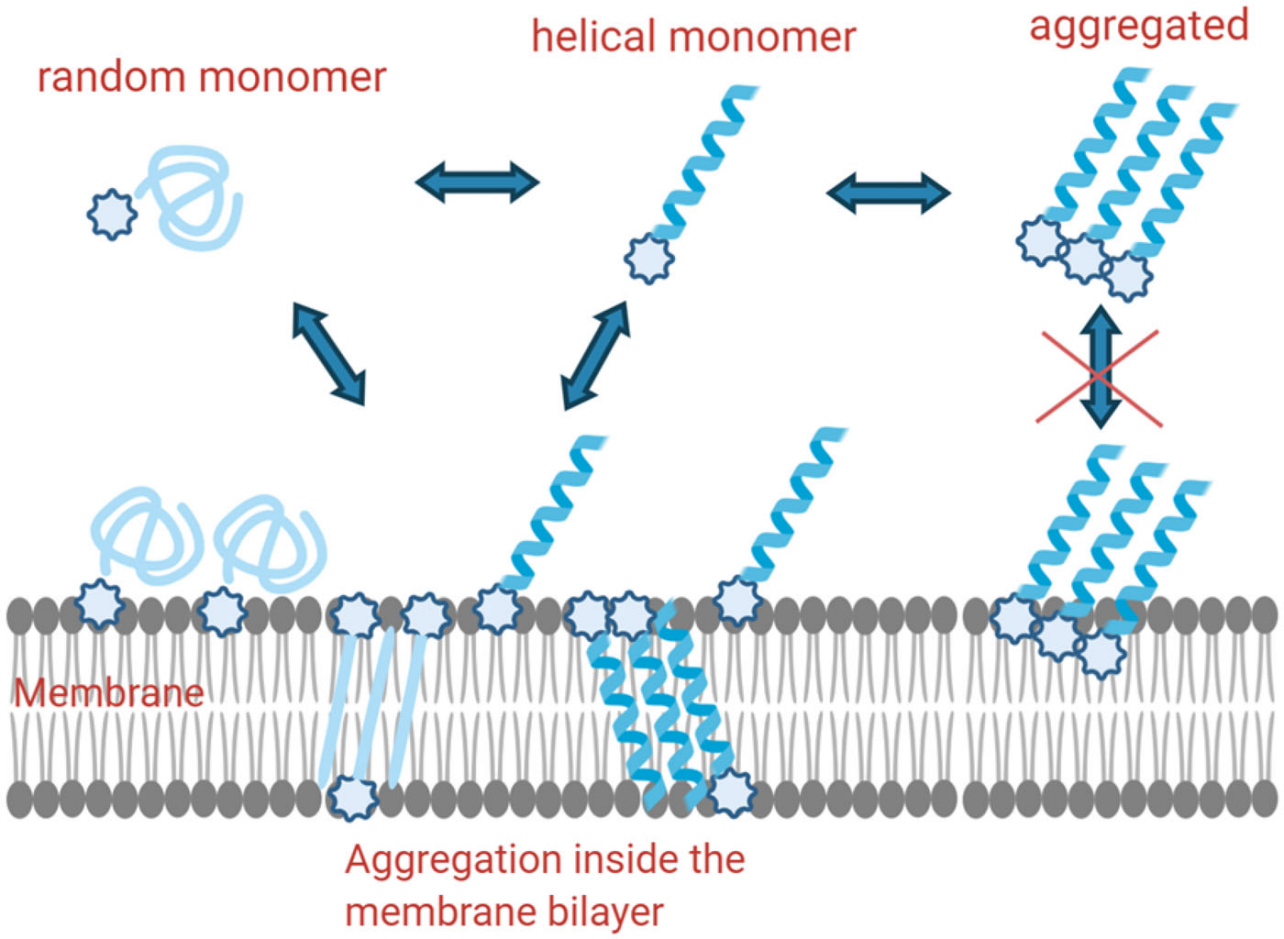
Schematic description of the hypothetical mechanisms involved in Temporin L coupled to lipid tags monomeric structures are involved in the membrane attachment, while self-aggregated peptides are present inside the membrane and both steps are influenced by the presence of lipid tags.

The analysis of the data obtained clearly shows that modification in the hydrophobicity and hydrophilicity may influence the self-aggregation in both water solution and in the bilayer. The most active peptide is monomeric in aqueous solution but is able to form molecular helical aggregates in the membrane environment of Gram-positive bacteria (Figure 6). In addition, it is also able to boost the membrane modifications which certainly are correlated to the affinity for the membrane and formation of pores.

The balance between hydrophobicity and hydrophilicity also helps to reduce haemolysis and toxicity. Interestingly, the same peptide (**C**) is the only one to show a higher activity against *K. pneumoniae* and *S. aureus*. The analysis of the data showed that also from a biophysical point of view the interaction of **C** with membrane mimicking Gram-negative bacteria is stronger compared to the other peptides. Nonetheless, it is interesting to note that the interaction is very different because the aggregation is less evident but the fluidity of the membrane changes more. The conformational change inside the membrane is clearly involving the presence of β-structures compared to helical aggregates in Gram-positive membranes. We may hypothesise that the mechanism of interaction with Gram-positive and negative bacteria is completely different, involving different secondary structures although both leading to leakage of the membranes.

Biological data on clinical strains further support the possible application of TL analogues such as those reported in this paper as novel AMPs able to help in the fight against antibiotic resistant bacteria.

## Supplementary Material

Supplemental Material
