# Supplementary material for: Novel temporin L antimicrobial peptides: promoting self-assembling by lipidic tags to tackle superbugs
Source: J Enzyme Inhib Med Chem. 2020 Sep 22;35(1):1751–64. doi: 10.1080/14756366.2020.1819258 (PMC7534258; doi:10.1080/14756366.2020.1819258)
Supplement: Supplemental Material [file IENZ_A_1819258_SM9825.pdf]

Supporting information

Novel Temporin L antimicrobial peptides: promoting self-assembling by lipidic tags to tackle superbugs

Rosa Bellavita,[a] Annarita Falanga,[b] Elisabetta Buommino,[a] Francesco Merlino,[a] Bruno Casciaro,[c] Floriana Cappiello,[d] Maria Luisa Mangoni,[d] Ettore Novellino,[a] Maria Rosaria Catania,[e] Rossella Paolillo,[e] Paolo Grieco[a]* and Stefania Galdiero[a]*

[a]Department of Pharmacy, University of Naples "Federico II", Naples 80131 (Italy); [b]Department of Agricultural Sciences, University of Naples "Federico II", Via Università 100, 80055 Portici, Italy; [c]Center for Life Nano Science@Sapienza, Italian Institute of Technology, Rome, Italy; [d]Department of Biochemical Sciences, Laboratory affiliated to Pasteur Institute Italia-Fondazione Cenci Bolognetti, Sapienza University of Rome, Rome 00185 (Italy); [e]Department of Molecular Medicine and Medical Biotechnologies, University of Naples "Federico II", 80131 Naples, Italy.
*Corresponding authors: stefania.galdiero@unina.it; paolo.grieco@unina.it

Table of contents

1. Synthetic path for the achievement of peptides **2A-5A**, **2B-5B, C, D, E** S2-3
2. Analytical data of peptides **2A-5A**, **2B-5B, C, D, E** (Table S1) S4
3. HPLC chromatograms of peptides **2A-5A**, **2B-5B, C, D, E** (Figures S3-13). S4-S9

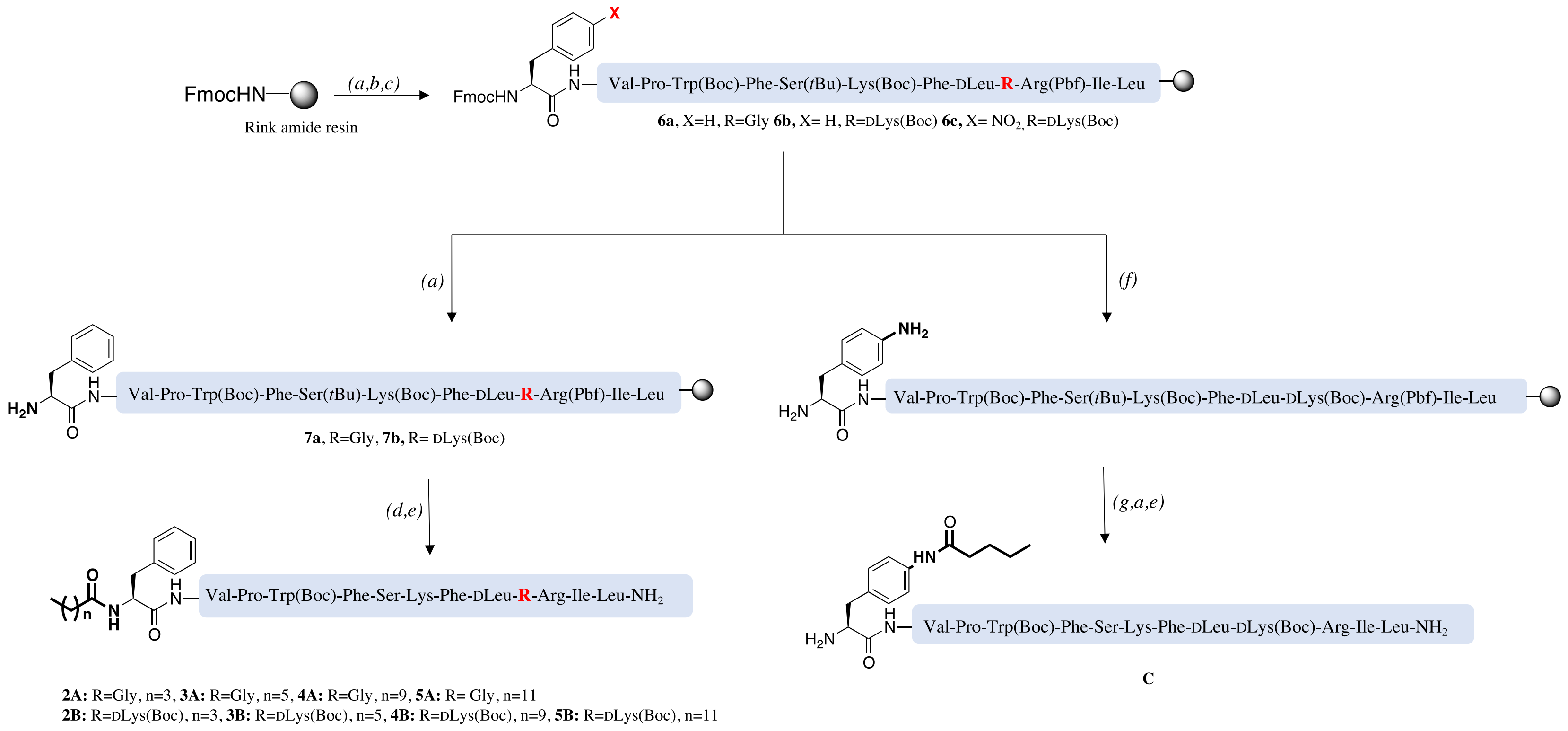

Scheme S1. (a) 20% piperidine in DMF, 0.5 + 1 min, ultrasonic irradiation; (b) Fmoc-Leu-OH (3 equiv), HBTU (3 equiv), HOBt (3equiv), DIEA (6 equiv), 1:1 DMF/DCM, 5 min, ultrasonic irradiation; (c) US-SPPS; (d) Fatty acid (3 equiv), HBTU (3 equiv), HOBt (3equiv), DIEA (6equiv), 1:1 DMF/DCM, 15 min, ultrasonic irradiation; (e) TFA/TIS/H_2O (95:2.5:2.5 v/v/v), 3h, rt; (f) $SnCl_2$ 1M in DMF, 12h, rt; (g) Valeric acid (3 equiv), HBTU (3 equiv), HOBt (3 equiv), DIEA (6 equiv), 2h, rt.

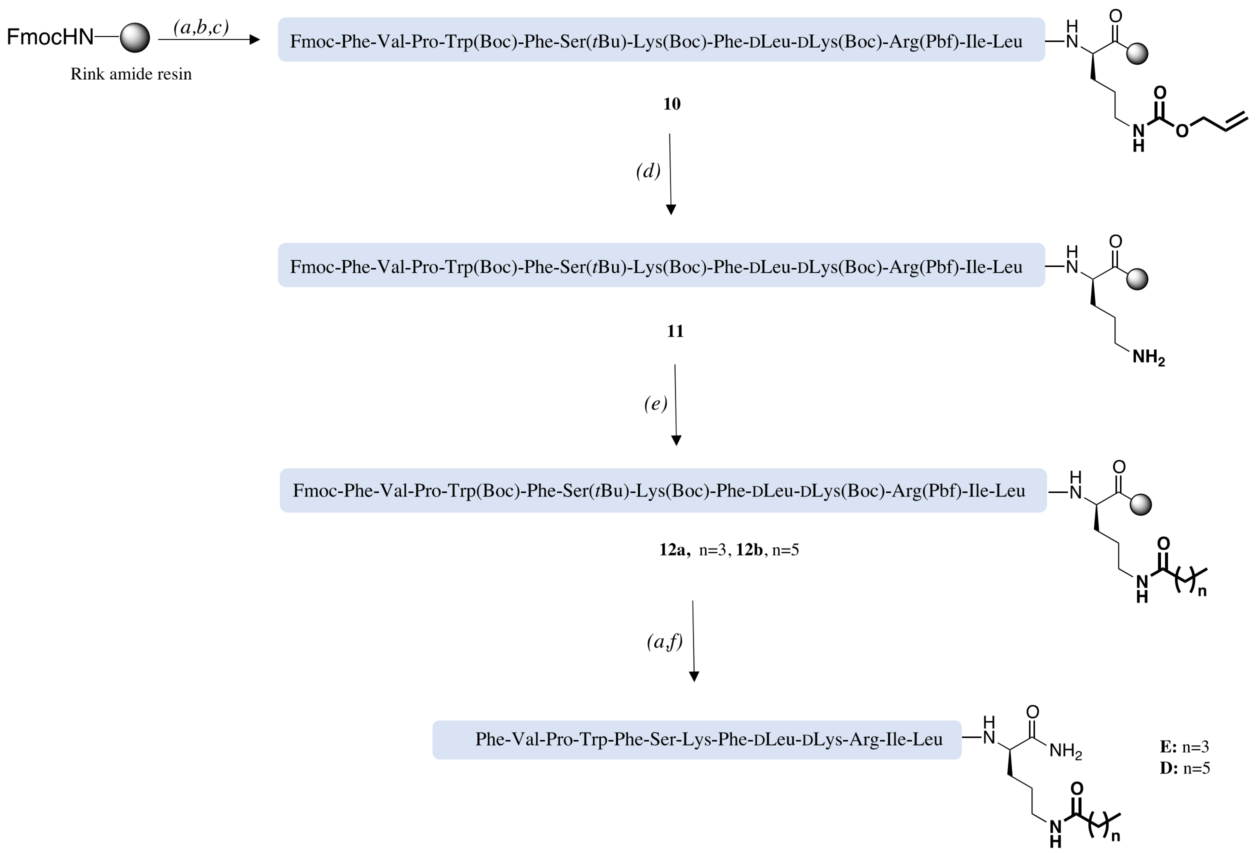

Scheme 2. (a) 20% piperidine in DMF, 0.5 + 1 min, ultrasonic irradiation; (b) Fmoc-Orn(Alloc)-OH (3 equiv), HBTU (3equiv), HOBt (3equiv), DIEA (6equiv), 1:1 DMF/DCM, 5 min, ultrasonic irradiation; (c) US-SPPS; (d) Pd(PPh_3)$_4$ (0.15 equiv), dimethylbarbituric acid (3equiv), DCM/DMF (3:2 v/v), 1h (x2), rt; (e) Fatty acid (3 equiv), HBTU (3 equiv), HOBt (3 equiv), DIEA (6 equiv), 1:1 DMF/DCM, 15 min, ultrasonic irradiation; (f) TFA/TIS/H_2O (95:2.5:2.5 v/v/v), 3h, rt.

	HRMS m/z	
**Compound**	**calcd.**	**obsd.**
**2A**	1694.0027	$[M+H/2]^+$= 847.5036
**3A**	1722.0340	$[M+H/2]^+$=861.5185
**4A**	1778.0966	$[M+H/2]^+$ = 889.551
**5A**	1806.1279	$[M+H/2]^+$= 903.5670
**2B**	1765.0762	$[M+2H/2]^+$=883.0450
**3B**	1793.1075	$[M+2H/2]^+$ = 897.0603
**4B**	1849.1701	$[M+H/2]^+$=925.0869
**5B**	1877.2014	$[M+2H/2]^+$= 939.1046
**C**	1780.0871	$[M+H/2]^+$= 890.5464
**D**	1879.1555	$[M+2H/2]^+$=940.0730
**E**	1907.1868	$[M+2H/2]^+$= 954.0976

Table S1. HRMS calculated and observed for peptides **2A-5A**, **2B-5B, C, D, E.**

HPLC chromatograms of peptides 2A-5A, 2B-5B, C, D, E

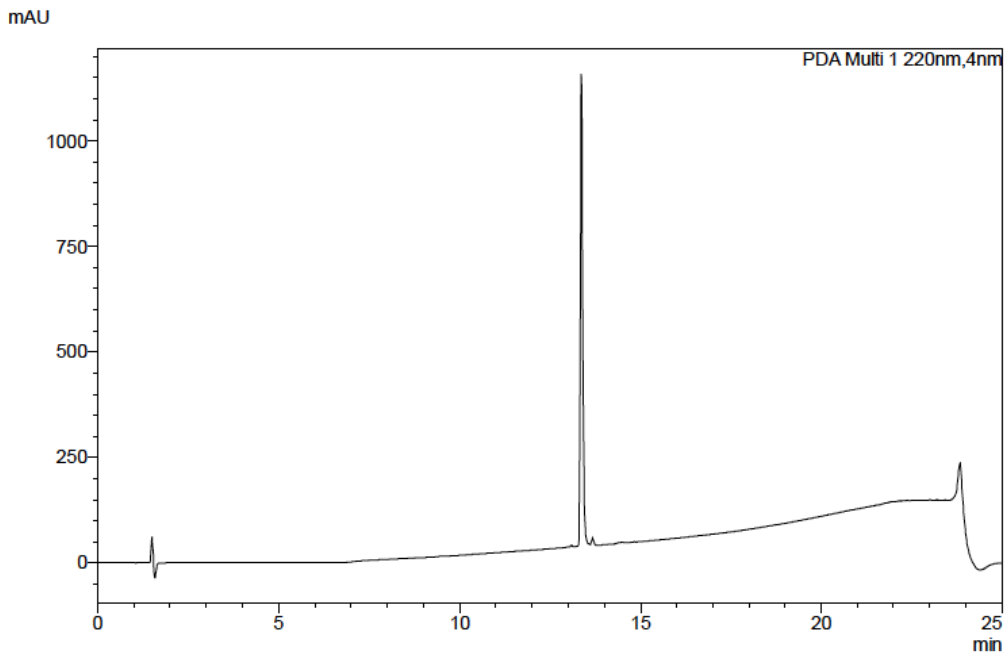

Figure S3. Chromatogram of **2A** obtained by an analytical HPLC (Shimadzu UFLC SPD-M20A/DGU-20A3R/LC-20AD) equipped with a Phenomenex Kinetex C18 column (150 mm × 4.6 mm, 5 µm, 100 Å), and monitored by UV detection at 220 nm. t_R: 13.4 min [linear gradient 10-90% MeCN (0.1% TFA) in H_2O (0.1% TFA) over 20 min, flow rate of 1 mL/min].

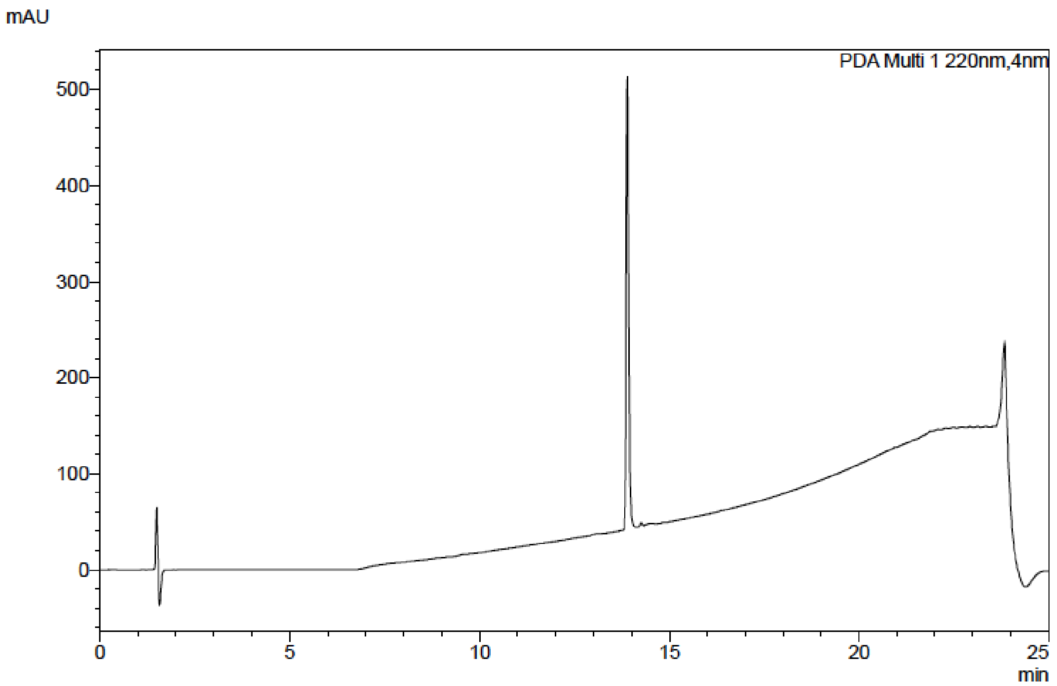

Figure S4. Chromatogram of **3A** obtained by an analytical HPLC (Shimadzu UFLC SPD-M20A/DGU-20A3R/LC-20AD) equipped with a Phenomenex Kinetex C18 column (150 mm × 4.6 mm, 5 µm, 100 Å), and monitored by UV detection at 220 nm. t_R: 13.9 min [linear gradient 10-90% MeCN (0.1% TFA) in H_2O (0.1% TFA) over 20 min, flow rate of 1 mL/min].

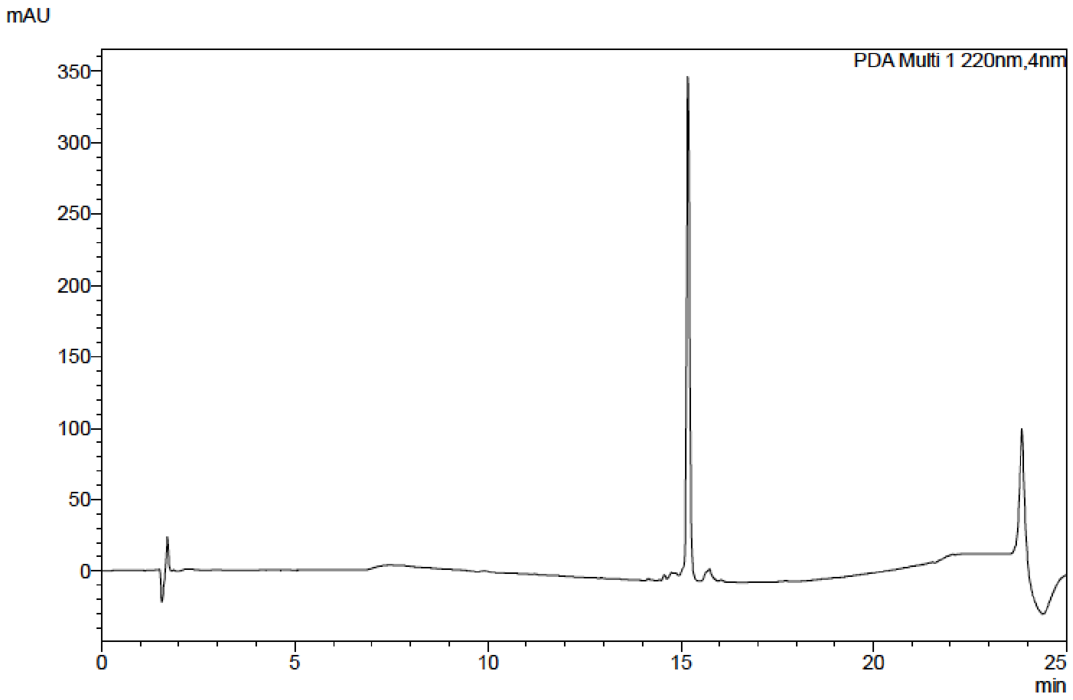

Figure S5. Chromatogram of **4A** obtained by an analytical HPLC (Shimadzu UFLC SPD-M20A/DGU-20A3R/LC-20AD) equipped with a Phenomenex Kinetex C18 column (150 mm × 4.6 mm, 5 µm, 100 Å), and monitored by UV detection at 220 nm. t_R: 15.8 min [linear gradient 10-90% MeCN (0.1% TFA) in H_2O (0.1% TFA) over 20 min, flow rate of 1 mL/min].

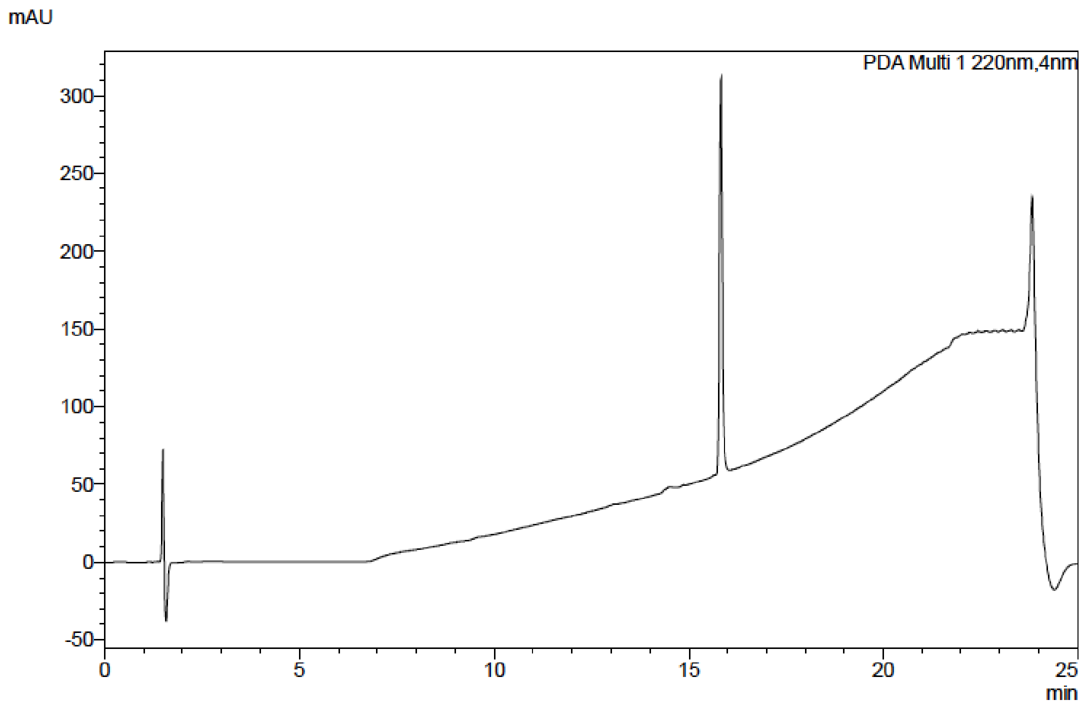

Figure S6. Chromatogram of **5A** obtained by an analytical HPLC (Shimadzu UFLC SPD-M20A/DGU-20A3R/LC-20AD) equipped with a Phenomenex Kinetex C18 column (150 mm × 4.6 mm, 5 µm, 100 Å), and monitored by UV detection at 220 nm. t_R: 15.2 min [linear gradient 10-90% MeCN (0.1% TFA) in H_2O (0.1% TFA) over 20 min, flow rate of 1 mL/min].

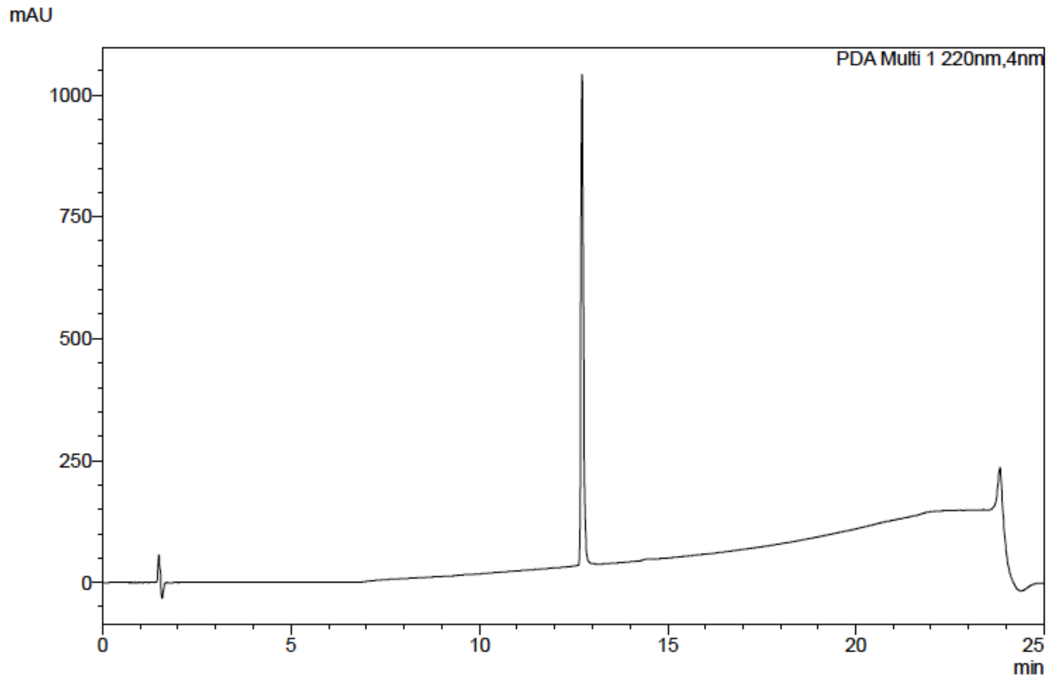

Figure S7. Chromatogram of **2B** obtained by an analytical HPLC (Shimadzu UFLC SPD-M20A/DGU-20A3R/LC-20AD) equipped with a Phenomenex Kinetex C18 column (150 mm × 4.6 mm, 5 µm, 100 Å), and monitored by UV detection at 220 nm. t_R: 12.7 min [linear gradient 10-90% MeCN (0.1% TFA) in H_2O (0.1% TFA) over 20 min, flow rate of 1 mL/min].

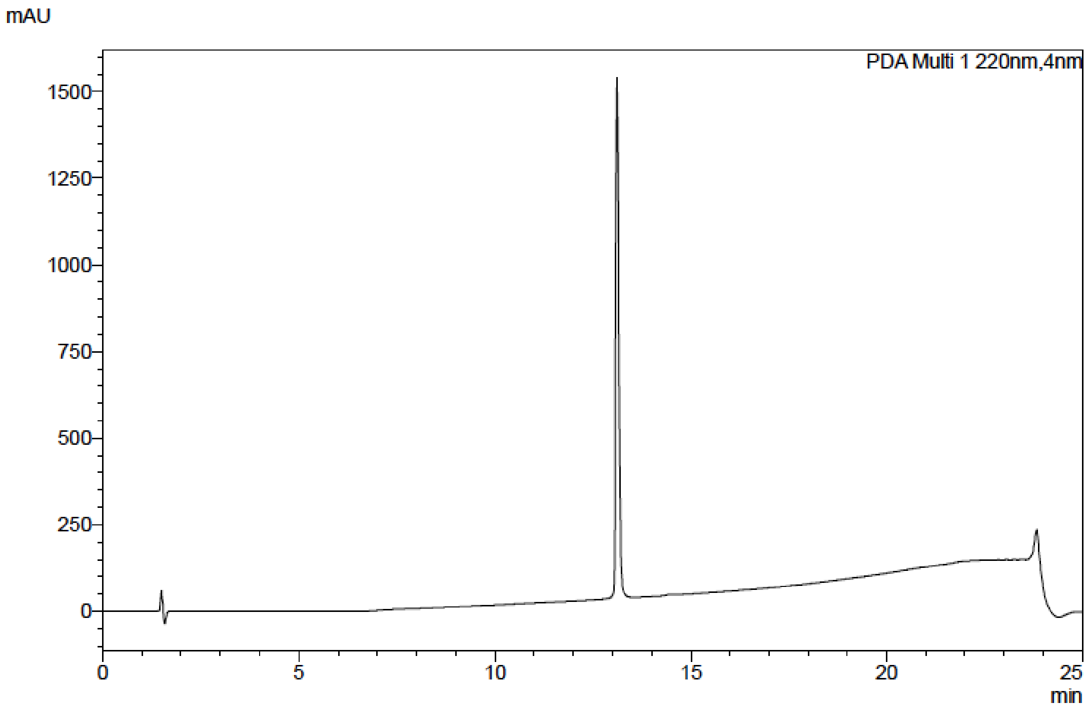

Figure S8. Chromatogram of **3B** obtained by an analytical HPLC (Shimadzu UFLC SPD-M20A/DGU-20A3R/LC-20AD) equipped with a Phenomenex Kinetex C18 column (150 mm × 4.6 mm, 5 µm, 100 Å), and monitored by UV detection at 220 nm. t_R: 13.1 min [linear gradient 10-90% MeCN (0.1% TFA) in H_2O (0.1% TFA) over 20 min, flow rate of 1 mL/min].

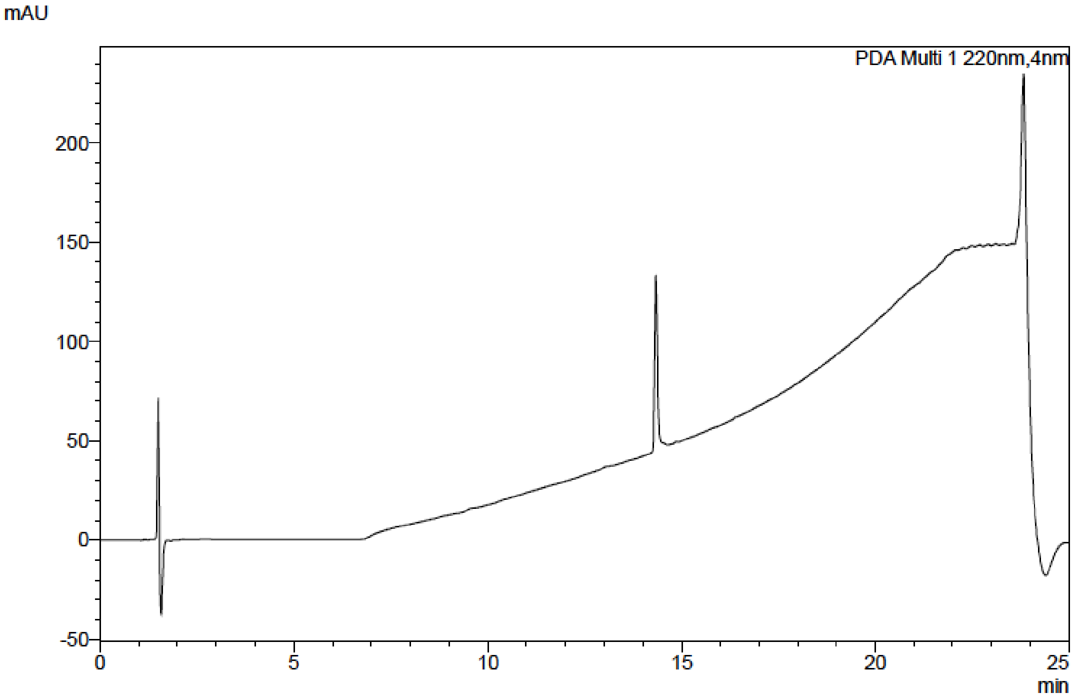

Figure S9. Chromatogram of **4B** obtained by an analytical HPLC (Shimadzu UFLC SPD-M20A/DGU-20A3R/LC-20AD) equipped with a Phenomenex Kinetex C18 column (150 mm × 4.6 mm, 5 µm, 100 Å), and monitored by UV detection at 220 nm. t_R: 14.9 min [linear gradient 10-90% MeCN (0.1% TFA) in H_2O (0.1% TFA) over 20 min, flow rate of 1 mL/min].

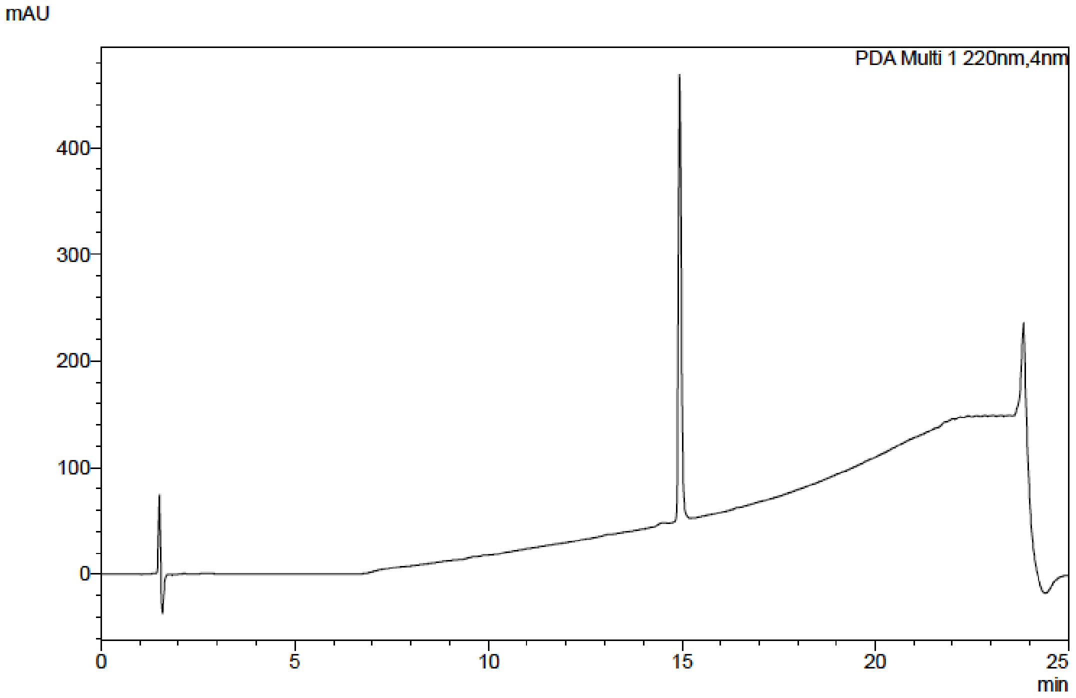

Figure S10. Chromatogram of **5B** obtained by an analytical HPLC (Shimadzu UFLC SPD-M20A/DGU-20A3R/LC-20AD) equipped with a Phenomenex Kinetex C18 column (150 mm × 4.6 mm, 5 µm, 100 Å), and monitored by UV detection at 220 nm. t_R: 14.3 min [linear gradient 10-90% MeCN (0.1% TFA) in H_2O (0.1% TFA) over 20 min, flow rate of 1 mL/min].

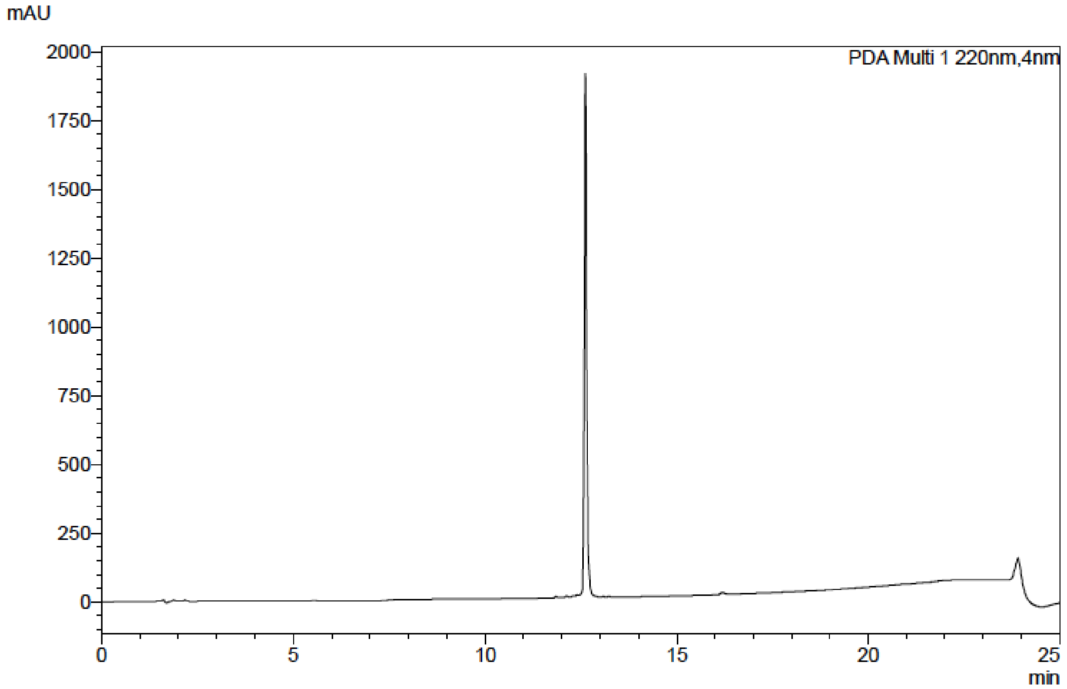

Figure S11. Chromatogram of **C** obtained by an analytical HPLC (Shimadzu UFLC SPD-M20A/DGU-20A3R/LC-20AD) equipped with a Phenomenex Kinetex C18 column (150 mm × 4.6 mm, 5 µm, 100 Å), and monitored by UV detection at 220 nm. t_R: 12.6 min [linear gradient 10-90% MeCN (0.1% TFA) in H_2O (0.1% TFA) over 20 min, flow rate of 1 mL/min].

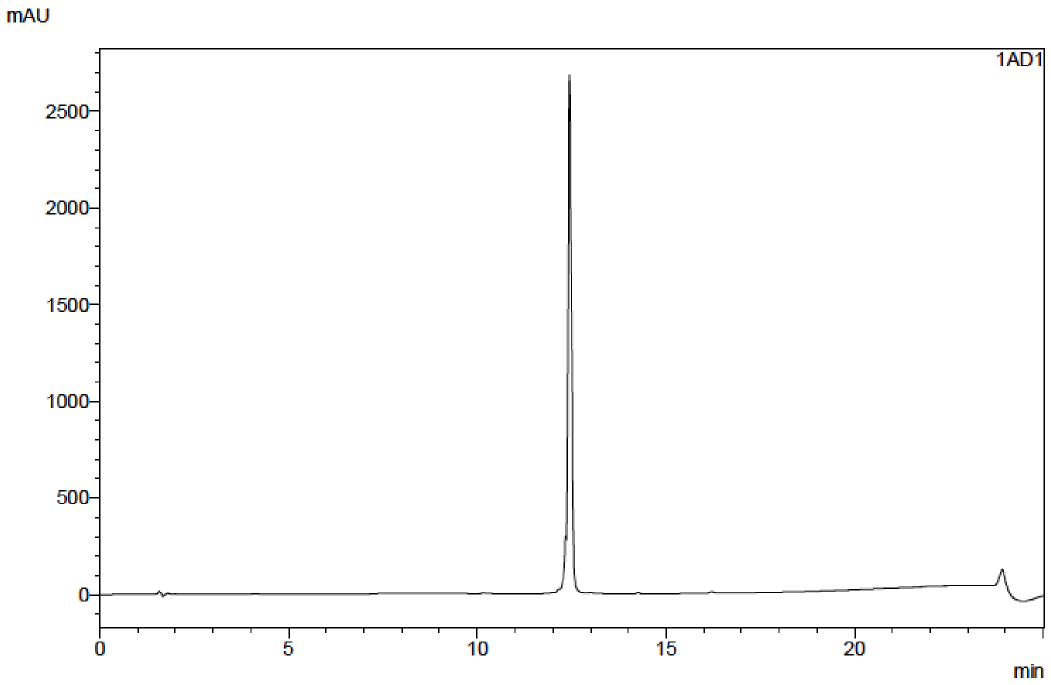

Figure S12. Chromatogram of **D** obtained by an analytical HPLC (Shimadzu UFLC SPD-M20A/DGU-20A3R/LC-20AD) equipped with a Phenomenex Kinetex C18 column (150 mm × 4.6 mm, 5 µm, 100 Å), and monitored by UV detection at 220 nm. t_R: 12.4 min [linear gradient 10-90% MeCN (0.1% TFA) in H_2O (0.1% TFA) over 20 min, flow rate of 1 mL/min].

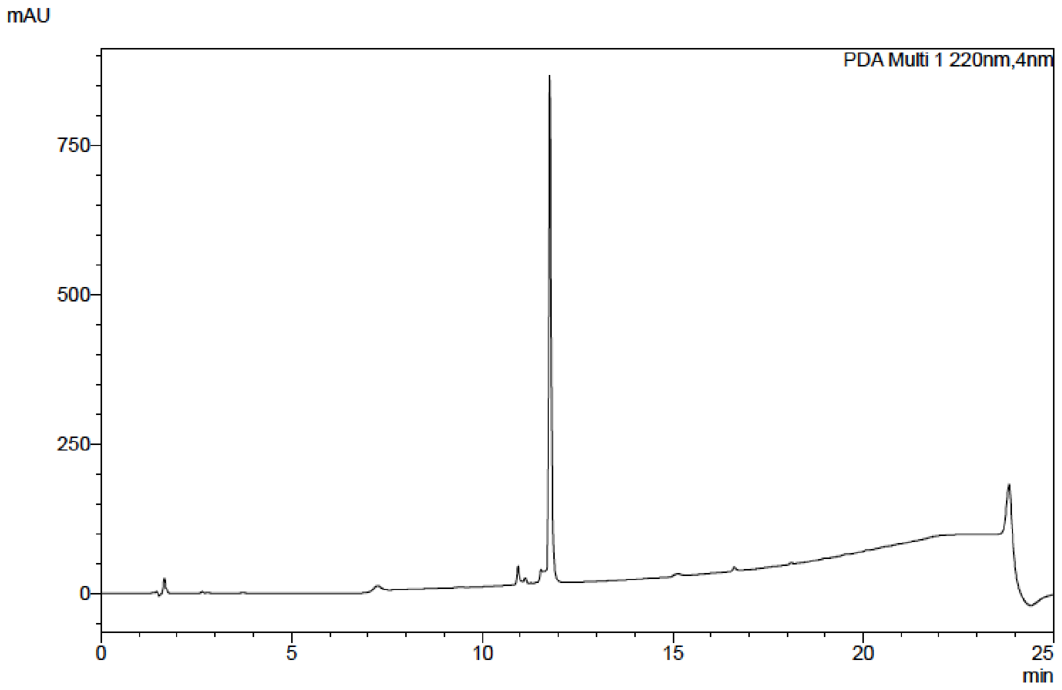

Figure S13. Chromatogram of **E** obtained by an analytical HPLC (Shimadzu UFLC SPD-M20A/DGU-20A3R/LC-20AD) equipped with a Phenomenex Kinetex C18 column (150 mm × 4.6 mm, 5 µm, 100 Å), and monitored by UV detection at 220 nm. t_R: 11.8 min [linear gradient 10-90% MeCN (0.1% TFA) in H_2O (0.1% TFA) over 20 min, flow rate of 1 mL/min].
